# Label leakage unmasked: a trustworthy-AI audit of autism screening models using the CLEAR-RD framework

**DOI:** 10.3389/fpubh.2026.1881829

**Published:** 2026-06-30

**Authors:** Boulbaba Ben Ammar, Walid Karamti

**Affiliations:** 1 Department of Computer Science, College of Computer, Qassim University, Buraydah, Saudi Arabia; 2 Data Engineering and Semantics Research Unit, Faculty of Sciences, University of Sfax, Sfax, Tunisia

**Keywords:** autism spectrum disorder, calibration, clinical machine learning, data quality, explainable AI, label leakage, personalized healthcare, Q-CHAT-10

## Abstract

Trustworthy pediatric screening AI needs more than accuracy: label validity, calibration, subgroup robustness, and clinical-scope limits. We propose Clear-Rd (*Clinical Leakage Evaluation and Audit Routine for Rule-Derived labels*), a five-stage framework—Configure, Lift, Evaluate, Audit, Report—and apply it to two public autism-screening datasets—Q-CHAT-10 toddler (*N* = 1054) and AQ-10 child/adolescent/adult (*N* = 6075)—merged into a unified cohort (*N* = 7129); prior studies report accuracies above 95%. Three configurations—*S*_1_ (full, with score), *S*_2_ (items, no score), and *S*_3_ (demographics only)—separate label leakage from demographic signal. A leakage audit confirms the Q-CHAT-10 label is exactly determined by score>3, the AQ-10 label approximately by score≥6. Sixteen models from three families (classical, deep-tabular, and prompt-structure simulators) are evaluated under 5-fold stratified cross-validation. On the leakage-free *S*_3_, Gradient Boosting attains ROC-AUC 0.766 ± 0.005 and all families converge within 0.04 ROC-AUC, indicating the binding constraint is the feature set, not model capacity. Calibration, subgroup-parity, and cost-sensitive analyses complete the audit. The results expose a gap between reported and clinically interpretable performance; Clear-Rd is a reusable template for rule-derived medical labels. No such model should be interpreted as predicting clinical autism diagnosis without external validation against gold-standard outcomes.

## 1 Introduction

Personalized healthcare is increasingly enabled by data-driven decision support that integrates clinical assessments, demographics, and digital phenotypes into individualized risk estimates (1, 2). For such systems to be safely translated into clinical practice, predictive performance alone is insufficient: trustworthy AI requires evidence of label validity, calibrated probabilities, subgroup robustness, and explicit limits on clinical scope (3–5). Accordingly, this work operationalizes trustworthiness through four concrete lenses that organize the entire audit: (i) *label validity*—whether the prediction target is an independent clinical outcome or a rule-derived artifact; (ii) *calibration*—whether predicted probabilities are reliable; (iii) *subgroup fairness*—whether performance and positive-prediction rates are equitable across demographic groups; and (iv) *clinical-scope limits*—an explicit statement of what the model may and may not be used for. A common but under-examined failure mode in this pipeline is the use of *rule-derived labels*, in which the prediction target is generated deterministically from a subset of the available features. When such labels are used as if they were independent clinical ground truth, conventional supervised-learning evaluations can produce inflated performance estimates and misleading claims of personalized clinical utility (6–9). A recent cross-disciplinary survey identified data leakage in at least 294 papers across 17 scientific fields and organized it into a taxonomy of eight types, underscoring that the problem is systemic rather than isolated (7).

Autism spectrum disorder (ASD) is a neurodevelopmental condition with an estimated prevalence of approximately 1 in 36 children in recent surveillance (10), for which early identification can support earlier intervention and better developmental outcomes (11, 12). In clinical practice, early identification frequently depends on short parent- or self-report screening instruments. The Quantitative Checklist for Autism in Toddlers, ten-item version (Q-CHAT-10) (13), and its older-age-group counterpart, the AQ-10, are two such instruments. Both have been widely used in studies on ASD-risk screening and both have associated public datasets released by Thabtah (14).

The two public datasets contain questionnaire responses, demographic metadata, a questionnaire score, and a binary ASD-traits label. Several machine-learning studies have treated these datasets as conventional supervised classification benchmarks and reported very high predictive performance (15–18). However, the target label in each dataset is not an independent clinical diagnosis: it is generated by thresholding the questionnaire score, and the score is computed from the questionnaire items. Consequently, any model trained using either the score or the questionnaire items can reconstruct, or closely approximate, the labeling rule directly.

This paper reframes the two datasets as a joint case study in trustworthy-AI evaluation for personalized pediatric and lifespan screening rather than as evidence for a deployable ASD-prediction system. We assemble a unified cohort by schema-harmonizing the toddler (*N* = 1, 054) and child/adolescent/adult (*N* = 6, 075) datasets into a single *N* = 7, 129 table, and we distinguish three experimental settings: a fully leaky configuration including the score, a questionnaire-items configuration excluding only the score, and a demographics-only configuration that removes the questionnaire entirely. This design lets us separate deterministic leakage from the residual predictive signal available from demographic metadata across the lifespan, and evaluate calibration, subgroup parity, and decision consequences under each configuration. Working with two datasets simultaneously lets us compare the leakage mechanism across both: the toddler rule is exact, while the AQ-10 rule is one-sided and approximate.

The study is guided by three explicit aims and their associated hypotheses. *Aim 1 (label validity):* to test whether the high reported accuracy on these datasets is attributable to label leakage; we hypothesize (H1) that the released label is a deterministic or near-deterministic function of the questionnaire score, so that any configuration retaining the score or items recovers it almost perfectly. *Aim 2 (residual signal):* to quantify the genuinely leakage-free predictive signal from demographics alone; we hypothesize (H2) that this signal is modest and, critically, *invariant to model capacity*, so that classical, deep-tabular, and prompt-structure model families converge to a common ceiling. *Aim 3 (trustworthiness audit):* to characterize the calibration, subgroup-parity, and decision behavior of the leakage-free model and to specify its clinical scope; we hypothesize (H3) that demographic predictors cannot match the screening instrument's own discriminative performance and therefore do not constitute a clinically deployable model.

The contributions are fourfold. First, we propose Clear-Rd (*Clinical Leakage Evaluation and Audit Routine for Rule-Derived labels*), a named, five-stage trustworthy-AI audit framework—Configure, Lift quantify, Evaluate decisions, Audit equity, Report scope—that is designed to be readily adaptable to other rule-derived medical labels. Second, we instantiate the framework on the unified Q-CHAT-10 / AQ-10 cohort, systematically exposing and quantifying both exact (toddler) and approximate (AQ-10) label leakage. Third, we provide a leakage-free demographics-only benchmark spanning three model families (classical machine learning, deep tabular networks, and prompt-structure simulators), and show that all three families converge to within 0.04 ROC-AUC of one another, indicating that the binding constraint is the feature set rather than the model class. Fourth, we clarify the clinical scope: the dataset label is a screening outcome, not a confirmed ASD diagnosis, and no evaluated model is clinically deployable without external validation against gold-standard assessment. The framework is designed to be applicable to other rule-derived screening labels common in personalized pediatric and preventive health pipelines.

## 2 Related work

### 2.1 Q-CHAT-10 dataset and screening-based learning

The Q-CHAT-10 instrument is a short parent-report screening tool for toddler ASD risk. The dataset released by Thabtah combines questionnaire responses with demographic metadata and has been used in multiple machine-learning studies for ASD-traits classification. Prior studies have applied decision trees, neural networks, and ensemble classifiers and have reported high classification accuracy. More recent work has begun to move beyond in-sample accuracy: studies have applied recursive feature selection while reporting discrimination metrics (AUROC) (19), and—most relevant to the present audit—have trained models on questionnaire-derived labels and then externally validated them against clinician-established diagnoses in an independent cohort (20). These developments are encouraging and align with the reporting practices that Clear-Rd formalizes; the gap we address is that such validation and calibration/fairness reporting remain the exception rather than the norm, and that the label-leakage mechanism itself has not previously been isolated across feature configurations on a unified cross-lifespan cohort.

A central limitation of this literature is that many studies treat the dataset as if its binary label were an independent clinical ground truth. In reality, the label is a screening-rule output derived from the questionnaire score. Therefore, performance obtained from the score or questionnaire items reflects agreement with a deterministic (or near-deterministic) rule rather than clinical diagnostic validity.

### 2.2 Limitations of existing approaches

Existing studies generally do not separate questionnaire-derived features from demographic features. This omission is important because the questionnaire score and items are not ordinary predictors: they are directly tied to the construction of the label, so a model using them reconstructs the label-generation mechanism rather than discovering an independent pattern associated with ASD risk. Moreover, the leakage pattern in the Q-CHAT-10 and AQ-10 public datasets has not been systematically isolated across separate feature configurations, evaluated on a unified cross-lifespan cohort, and benchmarked across model families on the same folds.

### 2.3 Label leakage in clinical machine learning

Label leakage occurs when predictors contain information that is derived from, or otherwise causally downstream of, the target variable. In clinical machine learning, leakage may arise through derived laboratory values, post-outcome variables, treatment decisions, or rule-generated labels. Such leakage can produce inflated performance estimates and misleading claims of clinical utility (6, 7, 21); in recent leakage taxonomies, a rule-generated label is a clear instance of the target variable leaking into the predictor set, because the score and items are inputs to the very rule that defines the label (7).

### 2.4 Trustworthy AI in healthcare

Biomedical artificial intelligence requires more than discrimination metrics. A credible model evaluation should consider interpretability, calibration, subgroup robustness, and the decision consequences of false negatives and false positives (1, 3). Methods such as SHAP (22), LIME (23), permutation importance, and partial-dependence plots can help inspect model behavior. Calibration metrics such as Brier score and expected calibration error assess probability reliability (24, 25), while statistical subgroup analyses and power analysis are necessary to avoid overinterpreting non-significant fairness tests (5, 26, 27). Reporting standards for clinical prediction models, including the recent TRIPOD+AI statement (4), increasingly require explicit description of the relationship between predictors and outcomes, confidence intervals, calibration, and subgroup analysis.

Several AI fairness and audit toolkits have been developed to support trustworthy-AI evaluation in healthcare settings, including Aequitas (28), IBM AI Fairness 360 (29), and Google PAIR (30). These toolkits offer general-purpose fairness metrics and bias-detection utilities applicable across domains. CLEAR-RD differs from these frameworks in three important respects: it is specifically designed for the setting in which the prediction target is a rule-derived label, it integrates label-leakage detection as its first mandatory stage (Stage C) before any performance evaluation is conducted, and it produces TRIPOD+AI-aligned clinical-scope artifacts rather than general fairness metrics. This targeted design makes CLEAR-RD directly applicable to screening instruments, risk-stratification scores, and wearable-derived labels with minimal adaptation, whereas general-purpose toolkits do not natively address the leakage patterns intrinsic to such datasets.

### 2.5 Trustworthy AI for personalized screening

Personalized healthcare AI aims to tailor screening, risk stratification, and intervention to the individual rather than the average patient, by integrating questionnaire responses, demographics, longitudinal history, and increasingly digital phenotypes into individualized risk estimates (1, 2). In this paradigm, the value of a model is not its aggregate accuracy but the reliability of the individual-level risk it communicates: a caregiver or clinician acts on a single predicted probability, so that probability must be calibrated, its drivers must be interpretable, and it must be equally trustworthy across demographic subgroups. Personalization therefore rests on label validity as a precondition—an individualized risk estimate is only meaningful if the target it predicts is a genuine clinical outcome rather than a deterministic recoding of the inputs. When the target is instead a rule-derived screening label, a model that appears to deliver “personalized” ASD risk is in fact reproducing a fixed scoring rule, and its apparent personalization is illusory. Several recent reviews emphasize that the gap between high in-sample accuracy and prospective clinical utility is mediated by data quality and label validity, especially when targets are surrogate outcomes derived from instruments rather than confirmed diagnoses (2, 4, 8, 9). The audit protocol developed here operationalizes these requirements for rule-derived screening labels, providing a template that can be reused across personalized pediatric and preventive-health pipelines.

### 2.6 Positioning of this work

Unlike prior studies that focus on maximizing classification accuracy on a single dataset, this work treats the Q-CHAT-10 and AQ-10 public datasets jointly, as a case study in clinical machine-learning validity for rule-derived labels. We isolate the leakage source on both datasets, evaluate a leakage-free demographics-only setting across three model families, and provide a calibration-, fairness-, and decision-aware trustworthy-AI audit. The “Prior Studies” column of Table 1 synthesizes the practices reported across representative Q-CHAT-10/AQ-10 machine-learning studies (15–18), which generally report headline accuracy without explicit leakage isolation, probability calibration, or formal subgroup-parity statistics; we emphasize that this characterizes the prevailing practice rather than the entire literature, and recent studies that incorporate feature selection with AUROC reporting (19) or external validation against clinical diagnoses (20) are acknowledged in Section 2. Table 1 summarizes the main differences between prior work and this study.

**Table 1:** Comparison of Clear-Rd with representative prior Q-CHAT-10/AQ-10 machine-learning studies across 12 methodological dimensions.

Aspect	Prior studies	Present work
Datasets	A single age-group dataset, or several modeled separately (15, 16)	Unified cohort: Q-CHAT-10 toddler ∪ AQ-10 child/adolescent/adult (*N* = 7129)
Target interpretation	Framed as ASD detection without an explicit clinical-scope limit (15, 16)	Explicitly defined as a screening outcome and a proxy label, not a clinical ASD diagnosis
Label leakage	Not isolated; questionnaire items and/or score used as predictors (15, 16)	Explicitly identified and quantified on both datasets: exact in toddler, approximate (one-sided) in AQ-10
Feature setting	Questionnaire items, score, and demographics used together (15, 16)	Three explicit configurations: *S*_1_ (full), *S*_2_ (items, no score), *S*_3_ (demographics only)
Model families	A single family (classical ML or neural networks) (15–17)	Three families compared on the same folds: 10 classical-ML, 2 deep-tabular, 4 prompt-structure simulators
Performance interpretation	Near-perfect accuracy reported as evidence of model effectiveness (15, 16)	Near-perfect performance interpreted as leakage artifact; non-leaky performance is modest and family-invariant
Uncertainty quantification	Point accuracy without confidence intervals (15, 16)	5-fold stratified cross-validation with per-fold mean ± standard deviation across all metrics
Statistical testing	No formal statistical model comparison (15, 16)	Friedman omnibus test + Nemenyi *post-hoc* critical-difference diagram
Fairness analysis	Subgroup/fairness analysis not reported (15, 16)	Per-subgroup performance disaggregation with SPD and disparate-impact statistics
Calibration	Probability calibration not reported (15, 16)	Brier score, log-loss, ECE, with Platt and isotonic calibration variants
Cost-sensitive operation	Cost-sensitive thresholding not reported (15, 16)	Threshold sweep across false-negative-to-false-positive cost ratios
Clinical claim	Early ASD detection or diagnosis implied (15, 16, 18)	Explicitly non-deployable without external clinical validation

## 3 The CLEAR-RD audit framework

### 3.1 Definition and scope

Clear-Rd (*Clinical Leakage Evaluation and Audit Routine for Rule-Derived labels*) is a generalizable, five-stage taxonomy for evaluating supervised models trained on labels of the form *y* = *g*(*X*_*R*_), where *g* is a deterministic rule and *X*_*R*_⊆*X* is a known subset of the feature vector. Such labels are pervasive in personalized health pipelines: questionnaire-thresholded screening instruments (e.g., Q-CHAT-10, PHQ-9, GAD-7), risk scores derived from electronic-health-record variables (e.g., CHA_2_DS_2_-VASc, MELD), and wearable-derived activity, sleep, or arrhythmia labels generated by fixed cut-offs. Clear-Rd is intended to be readily adaptable to any such setting, with only the labeling rule *g* and its input subset *X*_*R*_ instantiated per domain.

**Figure 1:**
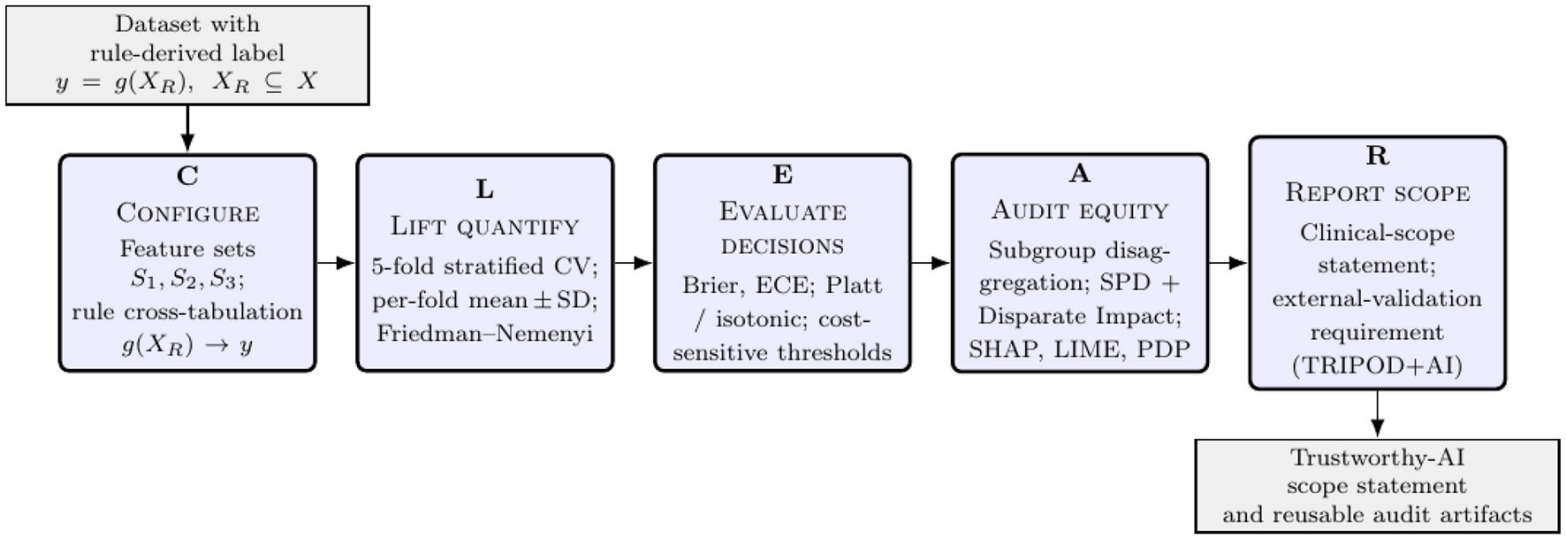
The Clear-Rd audit framework for rule-derived medical labels.

The five stages are summarized in Figure 1 and detailed below. Each stage produces a named artifact that can be archived alongside the model and reported in TRIPOD+AI (4) documentation.

### 3.2 Stage specifications

Stage C—Configure: Identify the labeling rule *g* and the feature subset *X*_*R*_ on which it is computed. Construct three feature configurations: *S*_full_ (all features, including *X*_*R*_ and any score derived from it), *S*_noscore_ (features minus the explicit score but retaining the rule's input variables), and *S*_nonleaky_ (features with *X*_*R*_ entirely removed). Report the rule cross-tabulation between *g*(*X*_*R*_) and *y* to make any deterministic dependence explicit.

Stage L—Lift quantify: Benchmark a panel of classifiers on each configuration with stratified *K*-fold cross-validation, reporting per-fold and mean±standard-deviation metrics for ROC-AUC, PR-AUC, F1, balanced accuracy, and Cohen's κ. Compare models with the Friedman omnibus test and visualize equivalence classes with a Nemenyi critical-difference diagram (31). Where appropriate, complement the classical-machine-learning panel with deep tabular networks [e.g., MLP-Emb, FT-Transformer (32, 33)] and *prompt-structure simulators*—deterministic, non-LLM procedures that reproduce only the input structure of zero-shot, few-shot, chain-of-thought, and ReAct prompting; this cross-family comparison probes whether the residual signal is unlocked by additional model capacity.

Stage E—Evaluate decisions: Audit probability calibration with the Brier score, log-loss, and expected calibration error (ECE), and compare uncalibrated, Platt-scaled (24), and isotonic-regressed predictors. Conduct a cost-sensitive threshold analysis across a range of false-negative-to-false-positive cost ratios and report stable operating regions, since false negatives and false positives carry asymmetric clinical costs in screening.

Stage A—Audit equity: Disaggregate test-fold performance by sensitive attributes (sex, jaundice, family ASD history, age group) and report per-subgroup accuracy, F1, balanced accuracy, and ROC-AUC, accompanied by group-level positive-prediction rates. For each binary attribute, report two complementary fairness statistics: the statistical parity difference SPD = *P*(Ŷ = 1∣*A* = *a*_1_)−*P*(Ŷ = 1∣*A* = *a*_2_) and the disparate-impact ratio DI = *P*(Ŷ = 1∣*A* = *a*_1_)/*P*(Ŷ = 1∣*A* = *a*_2_) (3, 26–29). Because subgroup sizes for sensitive attributes in publicly available screening datasets are typically small, parity statistics are reported as descriptive evidence rather than as hypothesis tests; we explicitly avoid framing non-significant outcomes as proof of fairness (5). Supplement with *post-hoc* explainability (SHAP (22), LIME (23), partial dependence, and permutation importance) treated as sanity checks rather than causal evidence.

Stage R—Report scope: Produce an explicit clinical-scope statement that distinguishes the rule-derived proxy label from a confirmed clinical outcome, names the gold-standard assessment that would be required for external validation, and aligns the audit outputs with TRIPOD+AI (4) reporting requirements. The statement is the deliverable that determines whether the model is suitable for clinical deployment, prospective study, or methodological use only.

The remainder of this section instantiates Clear-Rd on a unified cohort built from two public autism-screening datasets; Sections 4 and 5 report the corresponding outputs of stages L–A, and Section 8 delivers stage R.

### 3.3 Datasets and unified cohort

**Figure 2:**
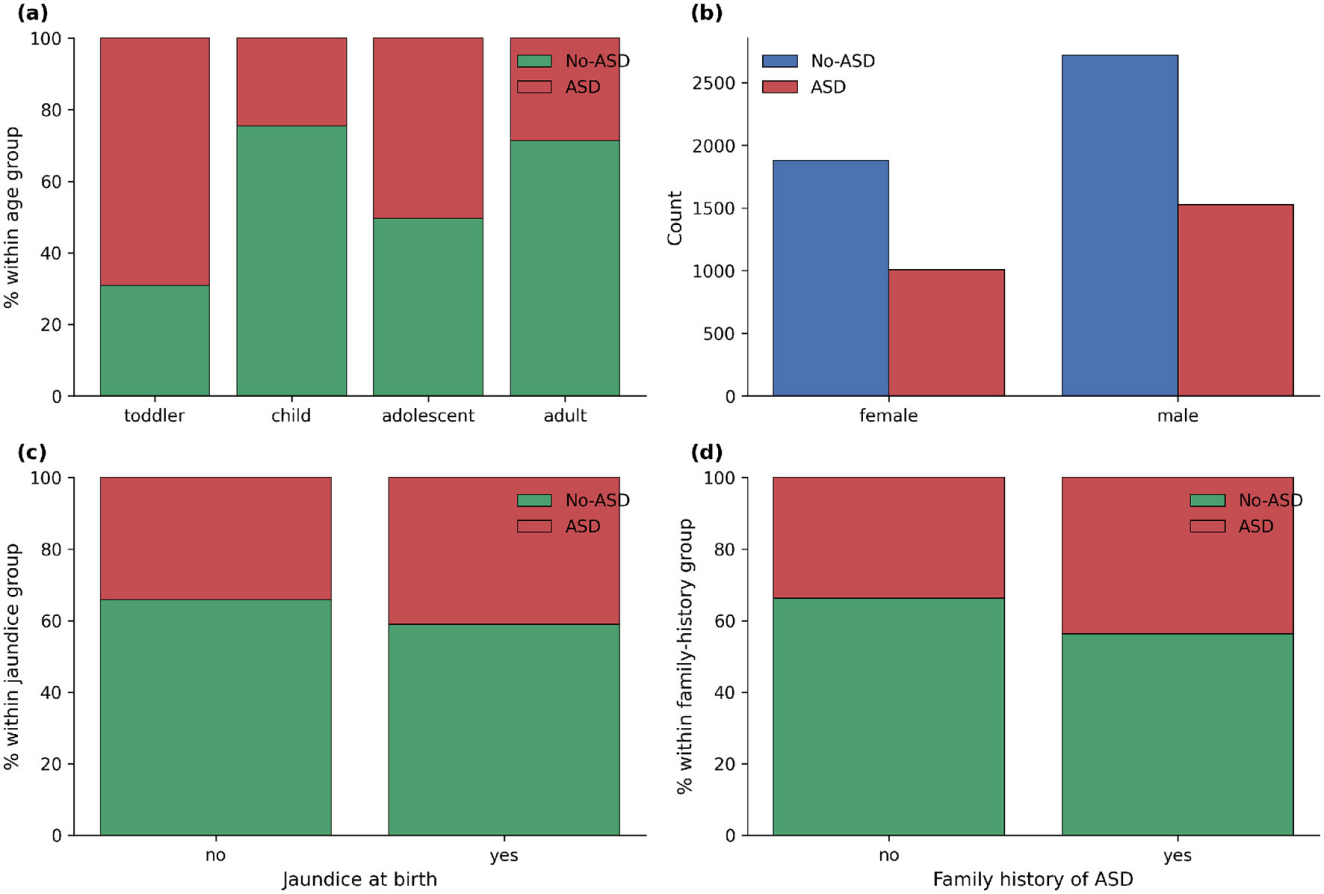
Unified-cohort overview: prevalence and class distribution after harmonization. **(a)** Percentage of ASD vs. No-ASD within each age group (toddler, child, adolescent, adult); **(b)** ASD vs. No-ASD counts by sex (female, male); **(c)** Percentage of ASD vs. No-ASD by jaundice-at-birth history (no, yes); **(d)** Percentage of ASD vs. No-ASD by family history of ASD (no, yes).

The unified cohort combines two publicly distributed autism-screening datasets released by Thabtah (14). The toddler dataset contains *N*_td_ = 1, 054 records with ten Q-CHAT-10 binary items (A1–A10), age in months, sex, jaundice history, family history of ASD, the Q-CHAT-10 total score, and a binary ASD-traits label (728 positive, 326 negative). The child/adolescent/adult dataset contains *N*_caa_ = 6, 075 records with ten AQ-10 binary items, age in years, sex, jaundice history, family history of ASD, and a binary screening label. Schema harmonization aligns the two sources on a common feature set: age in years (converting toddler months ÷12), sex, jaundice, family ASD history, an additional *age group* categorical (toddler / child / adolescent / adult), the ten questionnaire items (with the explicit caveat that the A1–A10 items represent different latent constructs in Q-CHAT-10 vs. AQ-10), and the labeling-rule score. Ethnicity and the test-administrator (“completed by”) variable are dropped because they are present only in the toddler dataset. The unified cohort comprises *N* = *N*_td_+*N*_caa_ = 7129 records. Figure 2 summarizes the class distributions across age group, sex, jaundice history, and family ASD history after harmonization.

### 3.4 Feature configurations

We evaluate three feature configurations. The first configuration, *S*_1_ (*full*), includes the screening score, the ten questionnaire items, and the demographic variables. The second configuration, *S*_2_ (*questionnaire*), removes the explicit score but retains the ten questionnaire items and demographics. The third configuration, *S*_3_ (*demographics-only*), removes the score and all questionnaire items, retaining only demographic features. Only *S*_3_ is leakage-free in the sense that no feature is an input to the deterministic labeling rule.

### 3.5 Models

Three model families are evaluated. The *classical machine-learning* family comprises ten estimators: a Dummy majority baseline, Logistic Regression, *k*-Nearest Neighbors, Decision Tree, support-vector machine with radial-basis-function kernel, Random Forest, Extra Trees, Gradient Boosting, XGBoost, and LightGBM. Categorical variables are one-hot encoded and numerical variables are standardized when required. All classifiers were trained with default hyperparameters in scikit-learn and compatible libraries (XGBoost, LightGBM); no hyperparameter search was performed.

The *deep tabular* family comprises two estimators: MLP-Emb, a multilayer perceptron with learned embeddings for categorical features, and FT-Transformer, a feature-tokenizer transformer for tabular data (32, 33). Both are trained with early stopping on a held-out fold.

#### 3.5.1 Prompt-structure simulators (deterministic, non-LLM)

##### 3.5.1.1 Terminology and scope

The four procedures described in this section are *not* large language models (LLMs) and do not perform any language-based or semantic reasoning. To avoid the ambiguity that terms such as “chain-of-thought” and “ReAct” can create—these are normally associated with generative LLMs, token-level reasoning traces, and external knowledge integration—we refer to them collectively as *prompt-structure simulators*. Each is a fully deterministic NumPy procedure that mirrors only the *computational skeleton* of a corresponding prompting strategy (a prior, a retrieval step, a confidence-sharpening step, or a tool-aggregation step), operating exclusively on the tabular features already available to every other model in the study. They contain no learned language parameters, consult no external corpus, and emit no natural-language output. Their purpose is narrow and methodological: to test whether the *structural ingredients* of popular prompting strategies—rather than the semantic capabilities of an actual LLM—recover any signal beyond the classical and deep-tabular families, while guaranteeing zero-cost, byte-identical reproducibility under a fixed seed and complete isolation from third-party API drift.

##### 3.5.1.2 The four simulators

**Figure 3:**
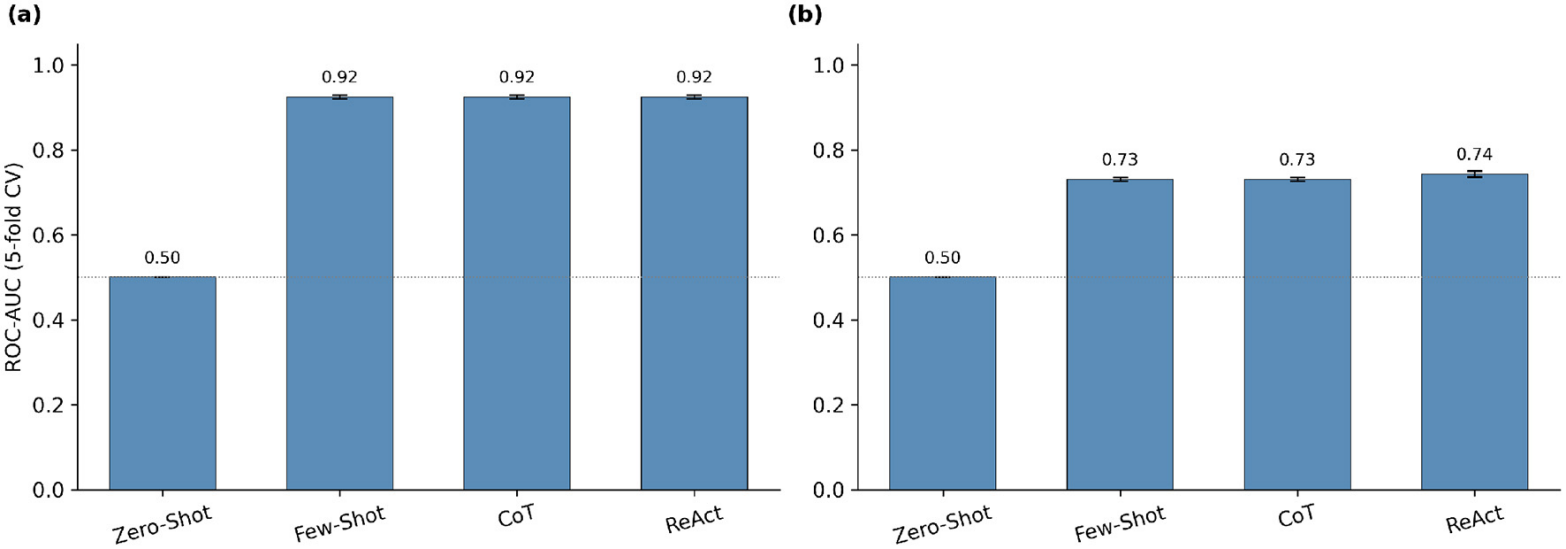
Prompt-structure simulators (P1–P4). **(a)** ROC-AUC on S2 ; **(b)** ROC-AUC on S3.

We evaluate four prompt-structure simulators (P1–P4), illustrated in Figure 3. Let the training fold be ${\left\{\left(x_{i},y_{i}\right)\right\}}_{i=1}^{n}$ with *y*_*i*_∈{0, 1}, and let a test instance be x_*_. All retrieval-based simulators use *k* = 8 neighbors under the Gower-style mixed-feature distance *d*(·, ·) (one-hot categorical components plus standardized numerical components), and all are evaluated on *S*_2_ and *S*_3_ only, since *S*_1_ is trivially solved by the score.

- P1—Zero-Shot returns the training-fold class prior, $\hat{p}\left(x_{*}\right)=ȳ=\frac{1}{n}\underset{i}{\sum }y_{i}$, for every test instance. It mirrors a zero-shot prompt that is given no worked example and can only fall back on the base rate; by construction it is uninformative (ROC-AUC ≈0.5) and serves as a floor.
- P2—Few-Shot is a similarity-weighted *k*-nearest-neighbor estimator, $\hat{p}\left(x_{*}\right)=\underset{j\in N_{k}}{\sum }w_{j}y_{j}/\underset{j\in N_{k}}{\sum }w_{j}$ with weights *w*_*j*_ = exp(−*d*(x_*_, *x*_*j*_)). The *k* retrieved neighbors play the role of the in-context exemplars that a few-shot prompt would place before the query.
- P3—Chain-of-Thought (structure only) uses the identical neighbor set and weighting as P2 but raises the weights to a sharpening exponent τ>1, $w_{j}\propto \exp{\left(-d\left(x_{*},x_{j}\right)\right)}^{\tau }$ (we use τ = 2), concentrating mass on the closest exemplars. This deterministically mimics only the *increased decisiveness* typical of step-by-step prompting; it involves no intermediate reasoning text whatsoever.
- P4—ReAct (structure only) interleaves “reasoning” and “acting” steps in the same purely arithmetic sense: it queries four fixed look-up “tools”—(1) age-group prevalence, (2) jaundice-history prevalence, (3) family-history prevalence, each returning the training-fold positive rate within the matching stratum, and (4) a top-*k* similar-cases retrieval identical to P2—and aggregates their outputs by averaging into a final probability. Intuitively, P4 mimics a clinician who, before deciding, consults a few reference prevalence statistics (“how common is a positive screen among toddlers? among children with a family history?”) and a handful of similar past cases, then combines them. Crucially, the “tools” are deterministic table look-ups over the training fold, not external knowledge sources.

##### 3.5.1.3 Relation to live LLMs

Because the simulators are deliberately stripped of semantic reasoning and external knowledge, their results bound the contribution of *prompt structure alone* and do not estimate what a genuine LLM might achieve. We address this directly in two ways. First, the central finding does not depend on the simulators: the classical and deep-tabular families—which include high-capacity models—also plateau at the same *S*_3_ ceiling, so the binding constraint is demonstrably the feature set, not the model class. Second, and more fundamentally, the prediction target on *S*_3_ is the questionnaire-derived label, which is itself a deterministic function of variables that are *absent* from *S*_3_; no model—however sophisticated, and including a live LLM with broad world knowledge—can recover information about the score from demographic features that do not encode it. A live LLM could plausibly add value only if it had access to richer inputs (e.g., free-text clinical notes) *and* those inputs correlated with the label; it could not raise the demographics-only ceiling for this particular proxy label. We therefore expect that any residual gain from a real LLM on *S*_3_ would be marginal and would reflect the feature space rather than reasoning capacity, and we leave a live-API replication as future work.

### 3.6 Evaluation protocol

All models are evaluated with stratified 5-fold cross-validation on the unified cohort. For each fold, the test partition is held out and all preprocessing, fitting, and prediction occur entirely on the training partition; per-fold metrics are then aggregated as mean±standard deviation across the five folds. The Friedman omnibus test is computed on per-fold ROC-AUC ranks across models (excluding Dummy), followed by a Nemenyi *post-hoc* critical-difference diagram. All random operations—stratified fold assignment, the deep-tabular train/validation split, and any internal model-side randomness—use a fixed random seed of 42 to ensure end-to-end determinism.

## 4 Results

### 4.1 Leakage demonstration

The labeling rule is fully deterministic for the Q-CHAT-10 toddler subset and approximately deterministic for the AQ-10 child/adolescent/adult subset. Because each score is computed from its dataset's questionnaire items, both *S*_1_ and *S*_2_ contain direct leakage in the toddler subset and substantial leakage in the AQ-10 subset. Table 2 reports the cross-tabulation of the screening-rule output against the released binary label for each dataset.

**Table 2:** Screening rule vs. released label: exact and approximate leakage.

Q-CHAT-10 toddler subset (*N* = 1054)		
	Class = Yes	Class = No
Score >3	728	0
Score ≤ 3	0	326
AQ-10 child/adolescent/adult subset (*N* = 6075)		
	Class = Yes	Class = No
Score ≥6	1,804	1,076
Score < 6	0	3,195

In the toddler subset the leakage is exact: every record satisfies the rule score>3⇔label = 1 without exception. In the AQ-10 subset the leakage is one-sided and approximate: the rule score≥6 produces no false negatives (every released positive label satisfies it) but does produce 1076 false positives, indicating that the released label is not exactly **1**[score≥6] but is strongly downstream of it. The two-dataset comparison provides a useful triangulation: the exact toddler rule rules out any subtler explanation of the near-perfect *S*_1_ performance reported in prior work, while the approximate AQ-10 rule shows that the same mechanism can operate even when the labeling rule is not perfectly recoverable from the score.

### 4.2 Cross-validated benchmark

**Figure 4:**
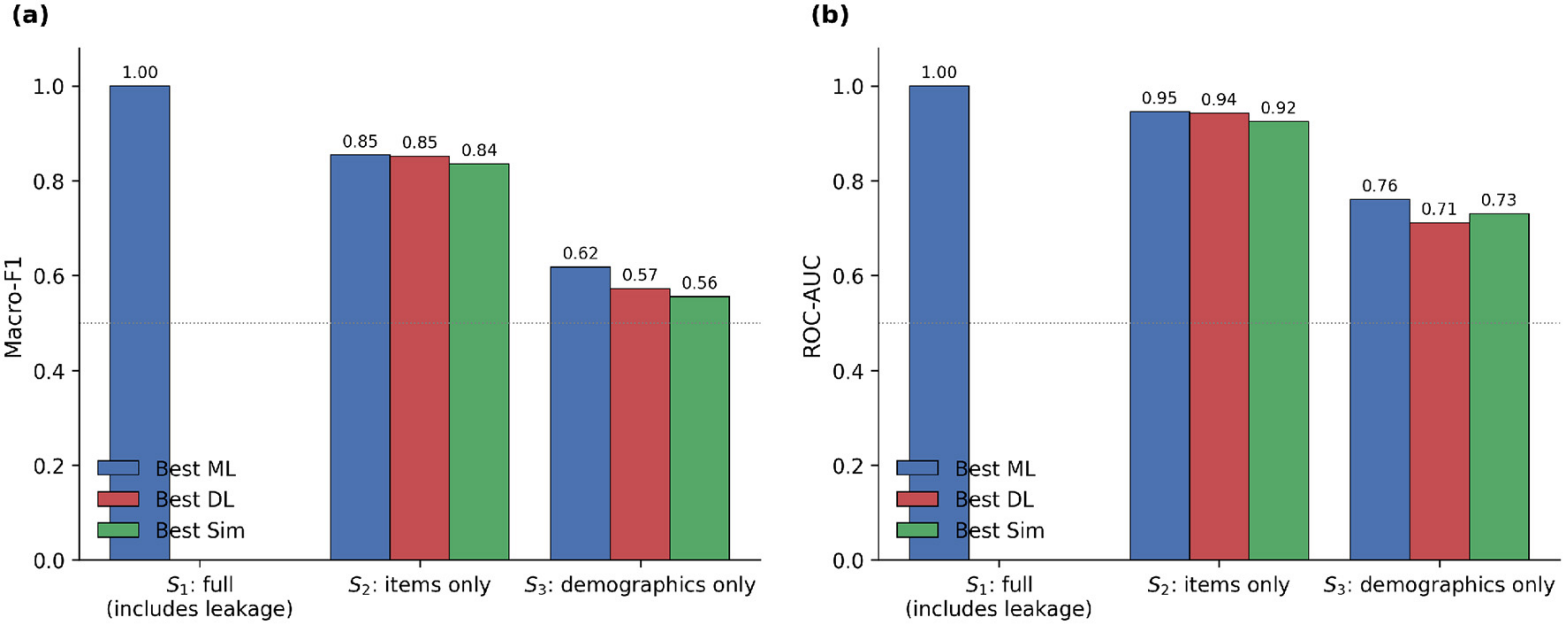
Best-model performance across the three feature configurations. **(a)** Macro-F1; **(b)** ROC-AUC.

In the leaky configurations, every non-trivial classifier reaches ROC-AUC of approximately 1.000 on *S*_1_ and 0.92–0.95 on *S*_2_. In the leakage-free *S*_3_ configuration, performance is substantially lower, indicating that the residual signal from demographic features alone is modest. Figure 4 illustrates the performance collapse from *S*_1_ to *S*_3_ across all three model families; Table 3 reports per-setup mean±standard deviation across the five stratified folds for the top classical-machine-learning models by *S*_3_ ROC-AUC.

**Table 3:** Five-fold CV performance of top ML models, *S*_1_–*S*_3_.

Configuration	Model	ROC-AUC	F1
*S*_1_	Random forest	1.000 ± 0.000	1.000 ± 0.000
*S*_1_	Gradient boosting	1.000 ± 0.000	1.000 ± 0.000
*S*_1_	XGBoost	1.000 ± 0.000	1.000 ± 0.000
*S*_2_	Gradient boosting	0.946 ± 0.004	0.855 ± 0.009
*S*_2_	XGBoost	0.944 ± 0.004	0.850 ± 0.010
*S*_2_	Random forest	0.937 ± 0.004	0.838 ± 0.005
*S*_3_	Gradient boosting	**0.766 ± 0.005**	0.523 ± 0.018
*S*_3_	XGBoost	0.762 ± 0.006	0.529 ± 0.009
*S*_3_	Decision tree	0.761 ± 0.003	0.619 ± 0.005
*S*_3_	LightGBM	0.753 ± 0.004	0.528 ± 0.009
*S*_3_	Random forest	0.748 ± 0.007	0.606 ± 0.010

Bold values indicate the best-performing result (highest ROC-AUC) reported in the table.

### 4.3 Cross-family comparison

**Figure 5:**
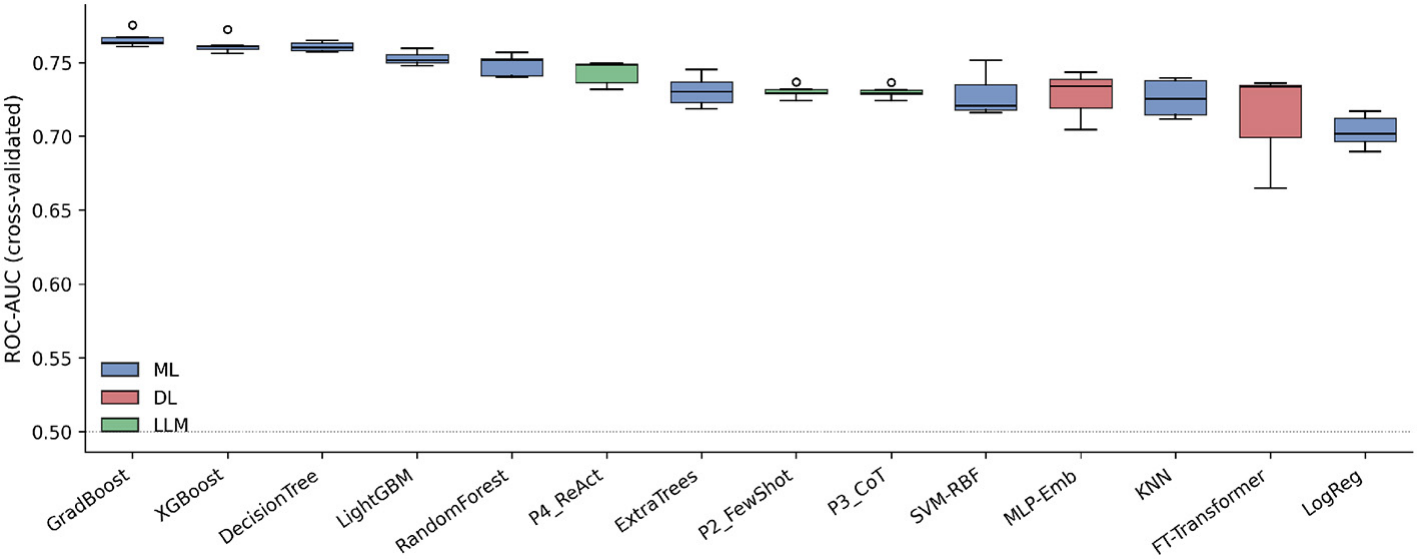
Per-fold ROC-AUC on leakage-free *S*_3_ by model family.

To probe whether the residual *S*_3_ signal is unlocked by additional model capacity, we compare the best classical-ML model against the best deep-tabular network and the best prompt-structure simulator. The per-fold ROC-AUC distribution across all evaluated models on *S*_3_ is shown in Figure 5, confirming narrow interquartile spreads (IQR < 0.02) for every model. Table 4 reports the top model in each family on *S*_2_ and *S*_3_. All three families converge to within 0.04 ROC-AUC on *S*_3_; deep-tabular and prompt-structure simulators do not improve upon Gradient Boosting on the leakage-free configuration. We interpret this convergence as evidence that the demographic feature set is the binding constraint, not the model class (34).

**Table 4:** Best model per family on *S*_2_ and *S*_3_.

Setup	Family/model	ROC-AUC	F1
*S*_2_	ML/gradient boosting	0.946 ± 0.003	0.855 ± 0.009
*S*_2_	DL/FT-transformer	0.943 ± 0.003	0.852 ± 0.004
*S*_2_	Sim / P2–P4 (tie)	0.925 ± 0.004	0.835 ± 0.006
*S*_3_	ML/gradient boosting	**0.766 ± 0.005**	0.523 ± 0.018
*S*_3_	DL/MLP-Emb	0.727 ± 0.017	0.559 ± 0.024
*S*_3_	Sim/P4 (ReAct)	0.743 ± 0.008	0.457 ± 0.011

Bold values indicate the best-performing result (highest ROC-AUC) reported in the table.

### 4.4 Statistical model comparison

**Figure 6:**
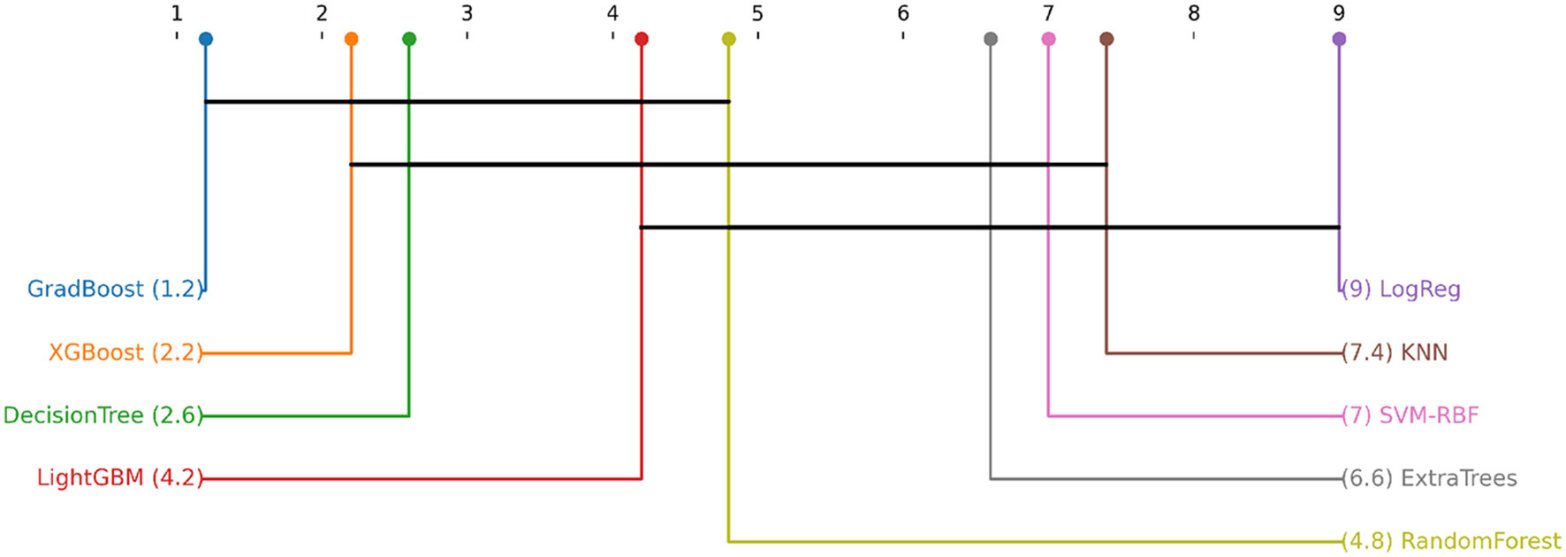
Nemenyi critical-difference diagram on *S*_3_ (α = 0.05).

We compare models using non-parametric procedures, which make no assumption about the distribution of the per-fold metrics and treat the five cross-validation folds as matched blocks across which all models are evaluated on identical partitions. The *Friedman omnibus test* ranks the *k* models within each fold (rank 1 = best ROC-AUC) and tests the null hypothesis that all models share the same mean rank, i.e., that any observed ordering is due to chance; its statistic is approximately χ^2^-distributed with *k*−1 degrees of freedom (31). Applied to the nine non-trivial classical-ML models (Dummy excluded; *k* = 9, *N* = 5 folds), it confirms significant rank differences across all three configurations: on *S*_1_ χ^2^(8) = 40.00, *p* = 3.20 × 10^−6^; on *S*_2_ χ^2^(8) = 36.75, *p* = 1.28 × 10^−5^; on *S*_3_ χ^2^(8) = 38.03, *p* = 7.44 × 10^−6^. Rejecting the omnibus null only establishes that the models are *not* all equivalent; to identify *which* pairs differ we apply the *Nemenyi post-hoc* test, in which two models differ significantly at level α if their mean ranks differ by more than the critical difference $\text{CD}=q_{\alpha }\sqrt{k\left(k+1\right)/\left(6N\right)}$, where *q*_α_ is the Studentized-range critical value (31). The resulting critical-difference diagram on *S*_3_ (Figure 6) places Gradient Boosting, XGBoost, and Decision Tree in the leading equivalence class and Logistic Regression in the lowest, with the leading group not significantly separated from the mid-ranked ensembles.

Two caveats on this analysis are important and motivate the complementary statistics below. First, with only *N* = 5 folds the Friedman/Nemenyi procedure has limited statistical power and a correspondingly wide critical difference, so the rank ordering should be read as indicative rather than definitive; consistent with this, the omnibus test detects *that* differences exist but the *post-hoc* test leaves most adjacent models statistically indistinguishable. Second, the omnibus test concerns relative ranks, not the absolute magnitude of differences that matters clinically. We therefore report ROC-AUC as the primary discrimination metric (35) and, because the unified cohort is class-imbalanced, also report PR-AUC and F1, since precision–recall summaries are more informative than ROC under imbalance (36). To attach uncertainty to the headline figures, we compute bias-corrected and accelerated bootstrap 95% confidence intervals (2,000 resamples of the held-out test-set predictions, *N* = 1426) for the best model in each family on *S*_3_. The held-out test ROC-AUCs and intervals are: Gradient Boosting (ML) = 0.759, 95% CI [0.734, 0.783]; the ReAct-structure simulator = 0.743, 95% CI [0.717, 0.768]; and MLP-Emb (DL) = 0.737, 95% CI [0.710, 0.765]. The three intervals overlap substantially, consistent with the cross-validated convergence reported in Section 4.3. To probe that convergence directly, we apply the DeLong test for two correlated ROC curves (37) on the common held-out set: Gradient Boosting vs. the ReAct-structure simulator yields ΔAUC = +0.016 (*p* = 0.075, not significant) and Gradient Boosting vs. MLP-Emb yields ΔAUC = +0.021 (*p* = 0.017). Even at the large held-out sample size (*N* = 1426), the Gradient Boosting–ReAct difference is not statistically significant, and although the Gradient Boosting–MLP-Emb difference does reach significance, its magnitude—about 0.02 ROC-AUC, well within the 0.04 cross-family band of Section 4.3—is practically negligible. The substantive conclusion is therefore unchanged: differences across model families are marginal in magnitude, so the demographic feature set, not model capacity, is the binding constraint on *S*_3_.

## 5 Trustworthy-AI audit

### 5.1 Explainability

**Figure 7:**
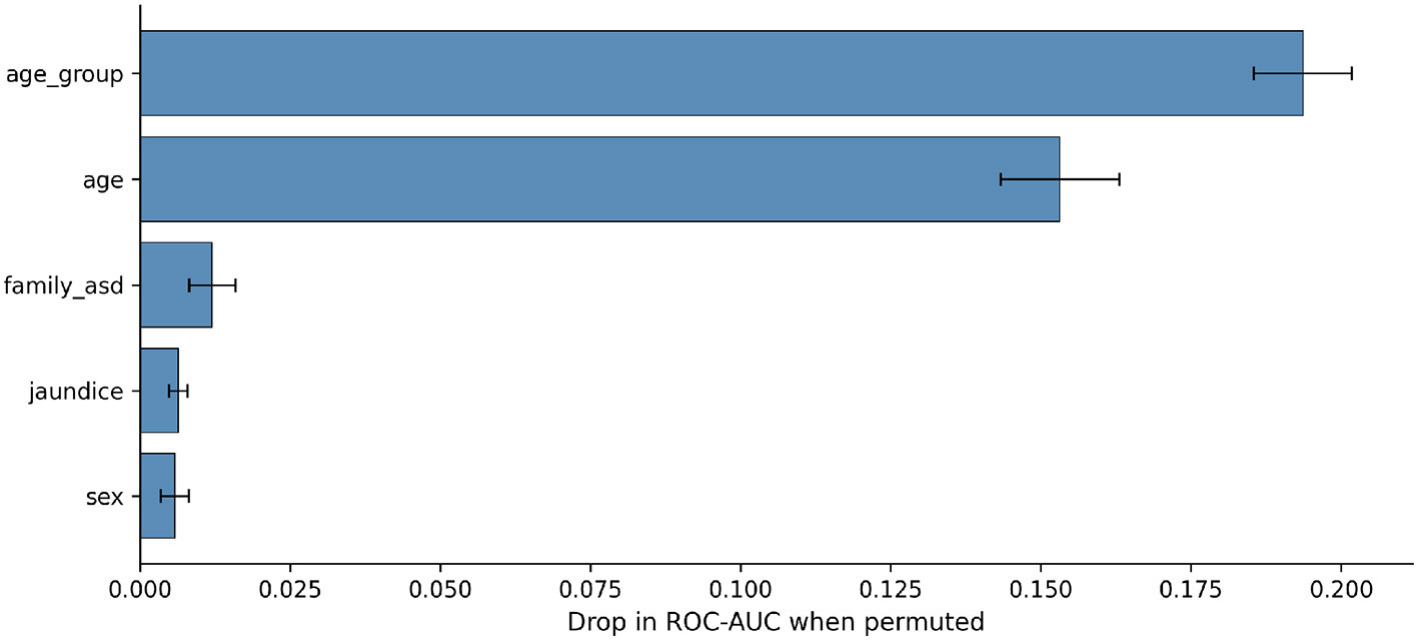
Permutation feature importance on leakage-free *S*_3_.

**Figure 8:**
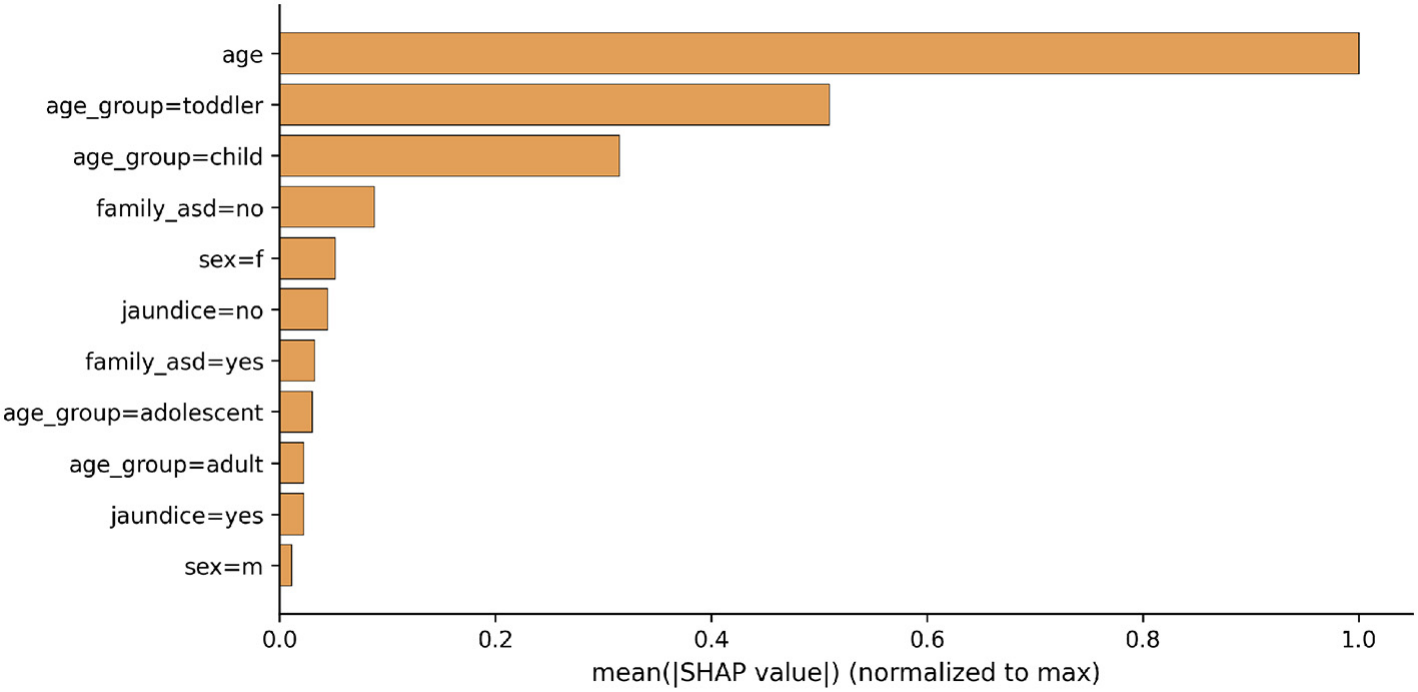
Global SHAP feature importance (S3, XGBoost).

**Figure 9:**
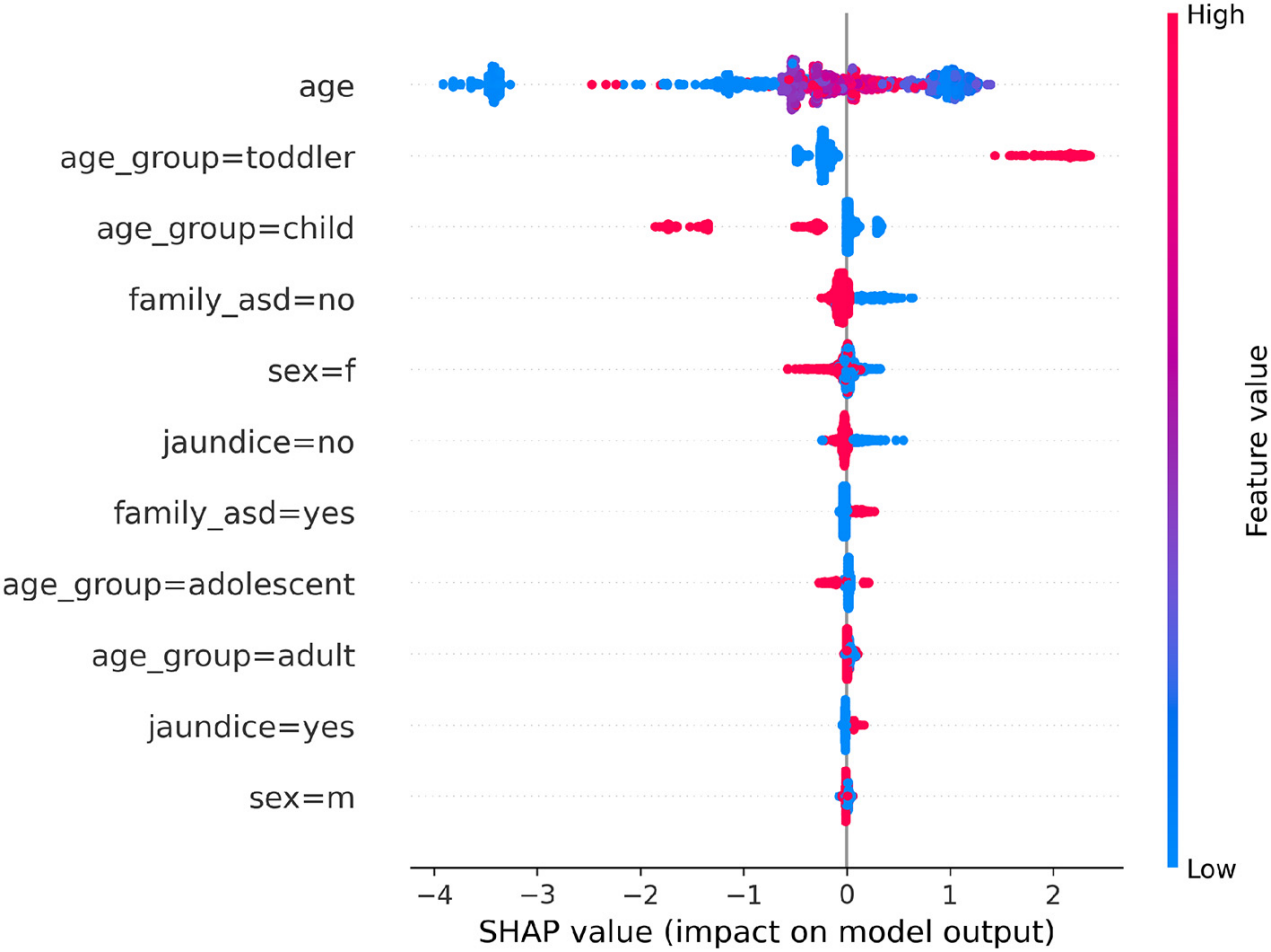
SHAP beeswarm plot (S3, XGBoost).

**Figure 10:**
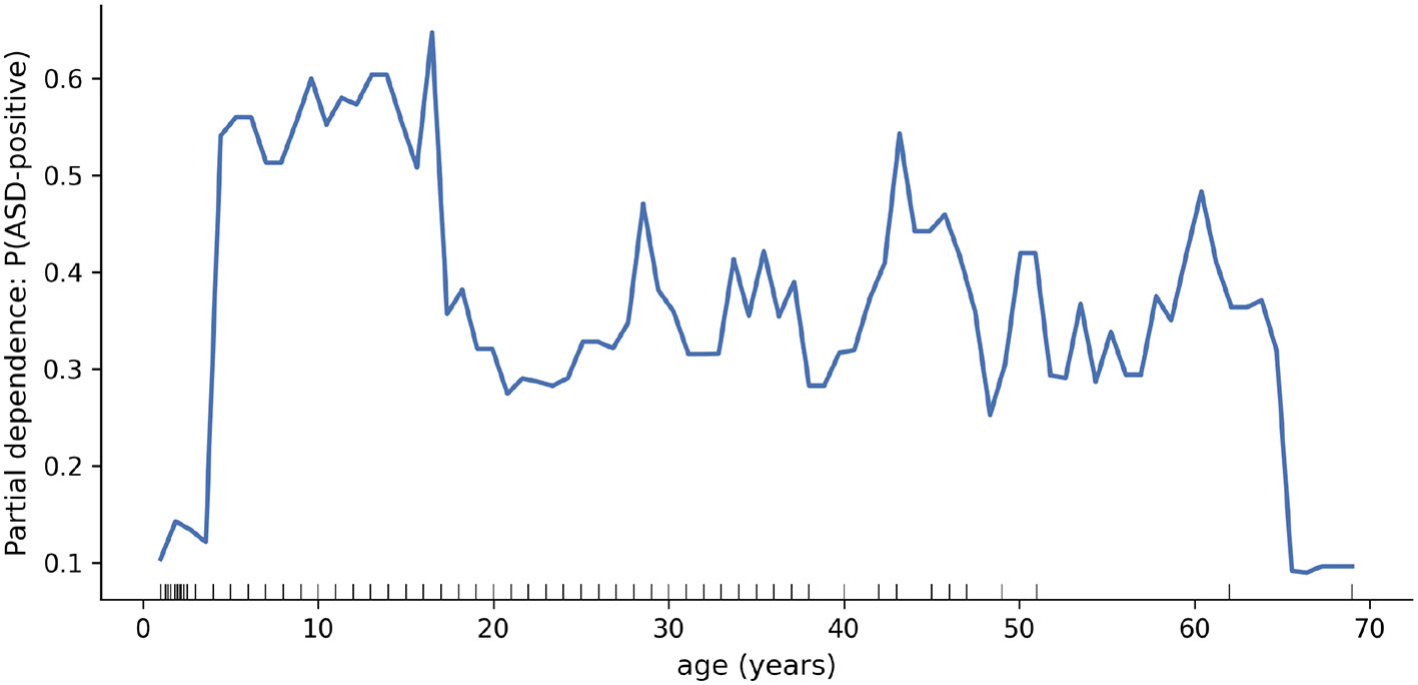
Partial-dependence of ASD-positive probability vs. age (*S*_3_).

**Figure 11:**
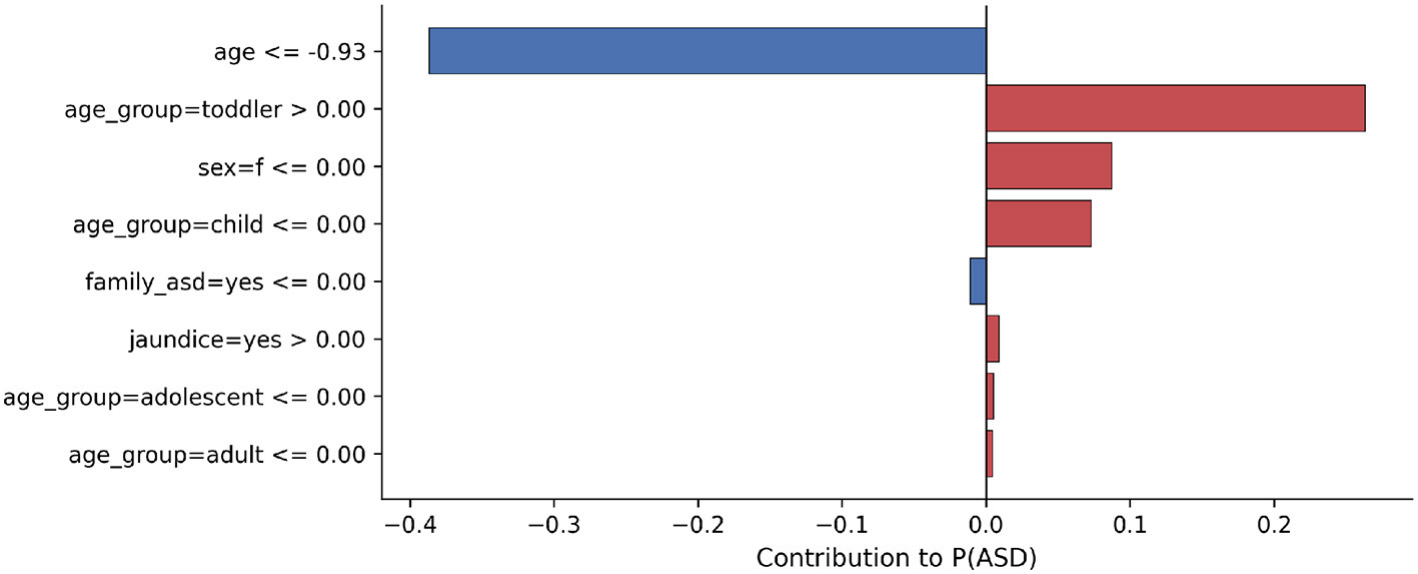
LIME explanation for a correctly predicted ASD-positive instance.

**Figure 12:**
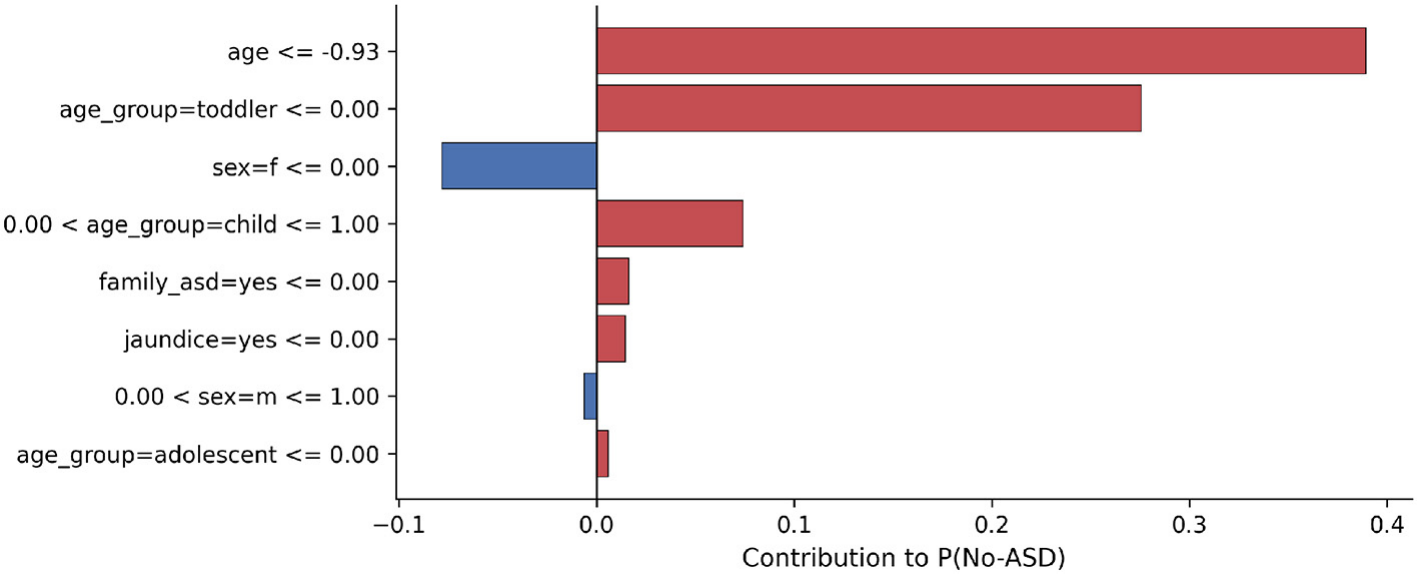
LIME explanation for a correctly predicted ASD-negative instance.

**Figure 13:**
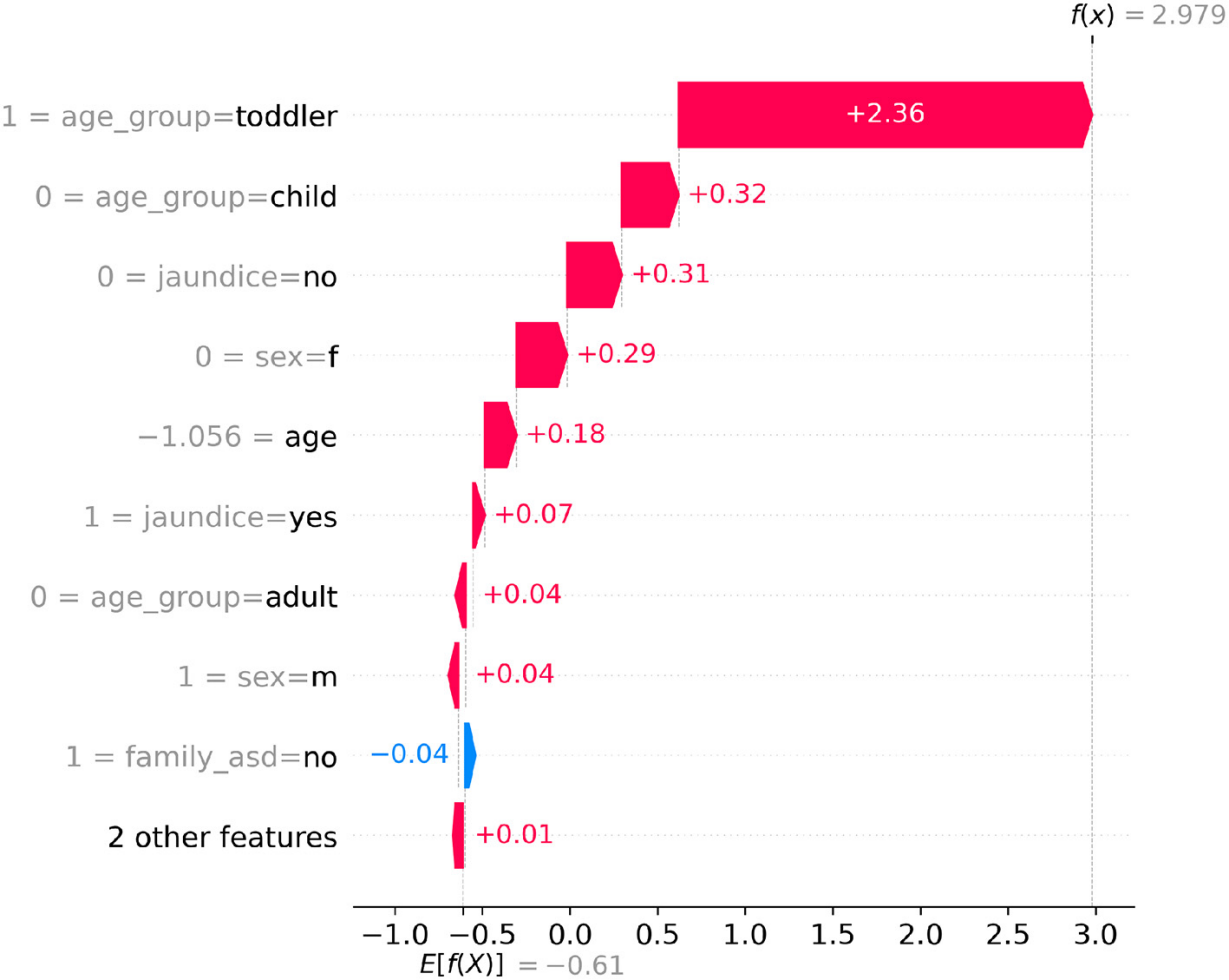
Local SHAP waterfall for a correctly classified ASD-positive instance (*S*_3_, XGBoost).

**Figure 14:**
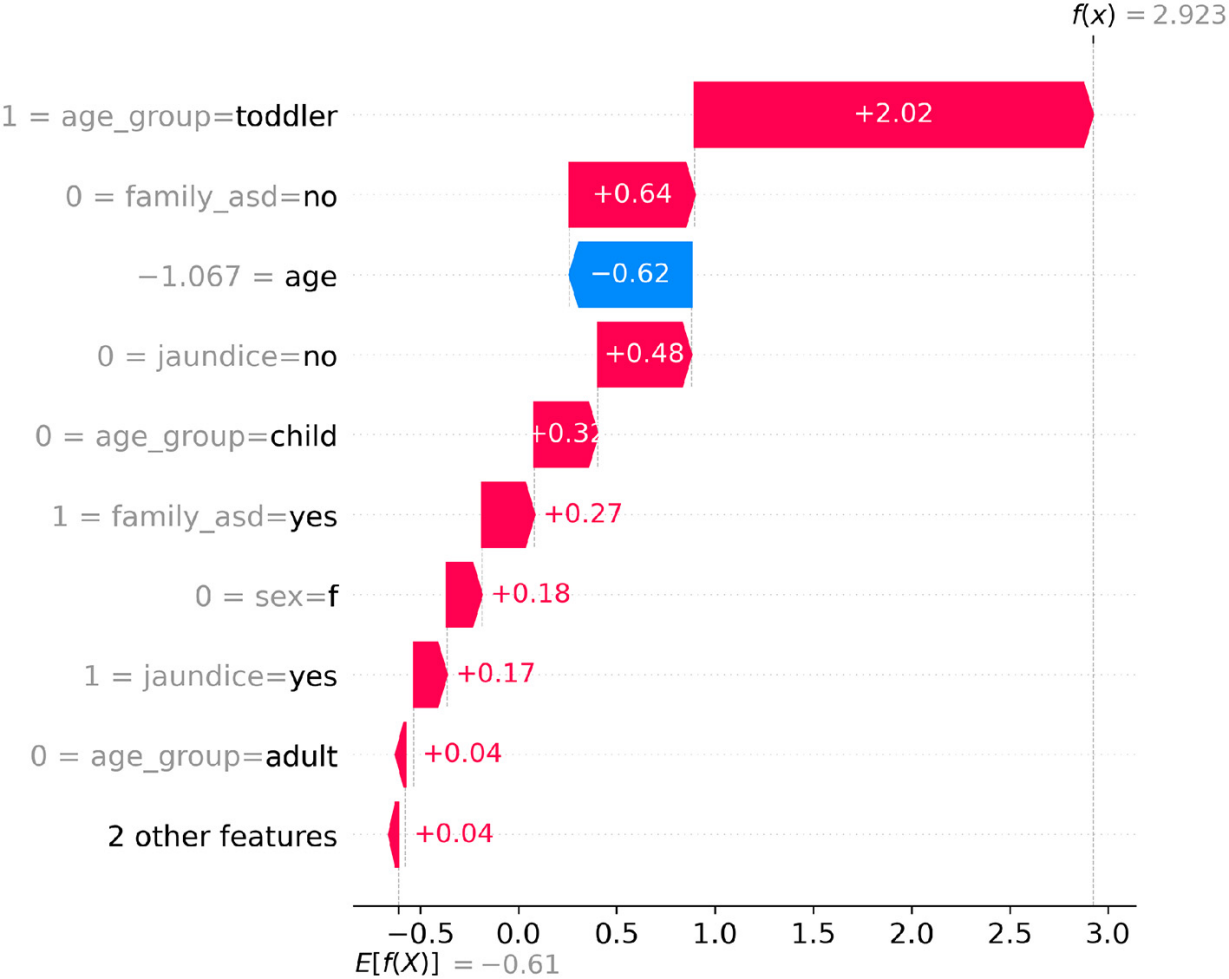
Local SHAP explanation for a misclassified instance.

The explainability audit focuses on the leakage-free *S*_3_ configuration. Permutation importance and SHAP agree that the two age features (age in years and age group) are the dominant predictors on the unified cohort, followed by family ASD history, jaundice history, and sex (Figure 7, Table 5). Global SHAP feature importance (Figure 8) confirms this ranking, with age and the toddler age-group indicator carrying the largest mean absolute SHAP values. The SHAP beeswarm plot (Figure 9) further shows that young age and the toddler group push predictions toward ASD-positive, while older ages push toward ASD-negative. The partial-dependence plot for age (Figure 10) reveals a non-monotone relationship: predicted ASD-positive probability peaks in early childhood and again in mid-adulthood, consistent with the merged age distribution of the two sub-cohorts. Local LIME explanations for a correctly classified ASD-positive instance (Figure 11) and a correctly classified ASD-negative instance (Figure 12) illustrate how young age and toddler-group membership dominate positive predictions, whereas older age suppresses them. Local SHAP waterfall plots for a correctly classified instance (Figure 13) and a misclassified instance (Figure 14) demonstrate that even strong toddler-group signals can be overridden by an atypical age value, explaining the residual errors of the model. Importantly, the dominance of the two age features must be interpreted with caution. Because the unified cohort merges a toddler-specific instrument (Q-CHAT-10) with a lifespan instrument (AQ-10), and the two source cohorts differ markedly in baseline ASD-trait prevalence (69% positive in the toddler subset vs. ≈30% in the AQ-10 subset), the age and age_group features partly encode *cohort membership and its associated screening base rate* rather than a biological effect of age—a structural artifact of harmonizing two convenience samples. Section 5.5 quantifies this contribution directly. Given the small feature set and the proxy-label nature of the target, these explanations should be interpreted as sanity checks on model behavior rather than as causal evidence of risk factors.

**Table 5:** Permutation feature importance on *S*_3_ (XGBoost).

Feature	Mean drop in ROC-AUC	SD
age_group	0.194	0.008
age	0.153	0.010
family_asd	0.012	0.004
jaundice	0.006	0.002
sex	0.006	0.002

### 5.2 Subgroup parity

**Figure 15:**
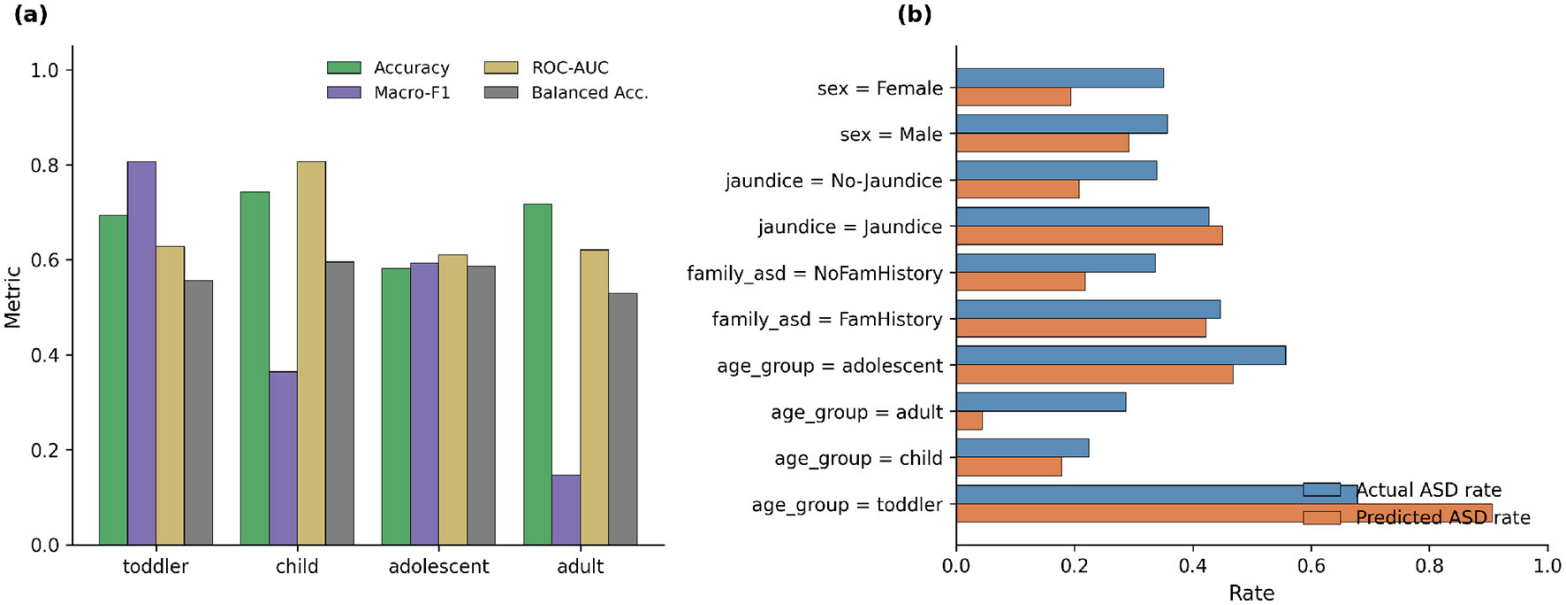
Per-subgroup performance on S3 by sex, jaundice, history, age. **(a)** Grouped bar chart of accuracy, macro-F1, ROC-AUC, and balanced accuracy by age group (toddler, child, adolescent, adult); **(b)** Actual vs. predicted ASD positive rate.

Subgroup analysis is conducted on the leakage-free *S*_3_ configuration with XGBoost as the operating model, disaggregating performance by sex, jaundice history, family ASD history, and age group. Per-subgroup positive-prediction rates and per-subgroup ROC-AUC are visualized in Figure 15 and reported in Table 6 together with two complementary fairness statistics. The *statistical parity difference* (SPD) is the absolute difference in the positive-prediction rate between two groups, where 0 denotes exact demographic parity: SPD = *P*(Ŷ = 1∣*A* = *a*_1_)−*P*(Ŷ = 1∣*A* = *a*_2_). The *disparate-impact ratio* (DI) is the ratio of those two rates, where 1 denotes parity and values below the conventional 0.8 (“four-fifths”) threshold are commonly flagged as evidence of adverse impact: DI = *P*(Ŷ = 1∣*A* = *a*_1_)/*P*(Ŷ = 1∣*A* = *a*_2_). The two attributes that are correlated with the rule-derived label by construction—jaundice history and family ASD history—show the largest gaps in predicted positive rate, consistent with the fact that both attributes raise the prior probability of a positive screening outcome in the underlying instrument. Because subgroup sizes for some attributes (e.g., female toddlers, family-history-positive adults) are small, the parity statistics are reported as descriptive evidence and not as hypothesis tests; we explicitly avoid framing the observed disparities as proof of either bias or fairness, and we note that prospective deployment would require external validation in a representative cohort.

**Table 6:** Subgroup parity statistics (SPD, DI) on *S*_3_.

Attribute	Group 1 (rate)	Group 2 (rate)	SPD	DI
Sex	Male (0.293)	Female (0.194)	+0.099	1.51
Jaundice	No (0.208)	Yes (0.451)	−0.243	0.46
Family ASD	No (0.218)	Yes (0.422)	−0.204	0.52

### 5.3 Calibration

**Figure 16:**
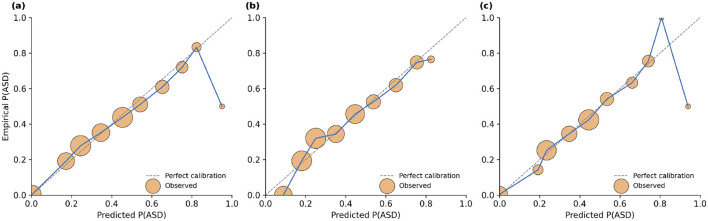
Reliability diagrams for XGBoost on S3 (three variants).

Probability calibration is audited for the leading operating model (XGBoost) on *S*_3_ using three variants: uncalibrated raw probabilities, Platt-scaled probabilities, and isotonic-regressed probabilities. Reliability diagrams (Figure 16) and the metrics in Table 7 show that the uncalibrated model is already well-calibrated on this configuration (ECE ≈0.02), and that isotonic regression marginally improves Brier, ECE, and log-loss while leaving ROC-AUC essentially unchanged. Platt scaling does *not* improve calibration on this dataset; it increases ECE relative to the uncalibrated model. ECE was computed using *M* = 10 equal-width bins following the standard convention of Guo et al. (25). We interpret these results as evidence that recalibration is not automatically beneficial in this setting and that calibration auditing should be performed as a routine Clear-Rd stage-E artifact rather than assumed.

**Table 7:** Calibration metrics for XGBoost on *S*_3_.

Model	Brier	ECE	Log-loss	ROC-AUC
XGBoost (uncalibrated)	0.186	0.021	0.538	0.758
XGBoost (Platt)	0.187	0.033	0.549	0.760
XGBoost (isotonic)	0.186	0.016	0.536	0.760

### 5.4 Cost-sensitive threshold analysis

**Figure 17:**
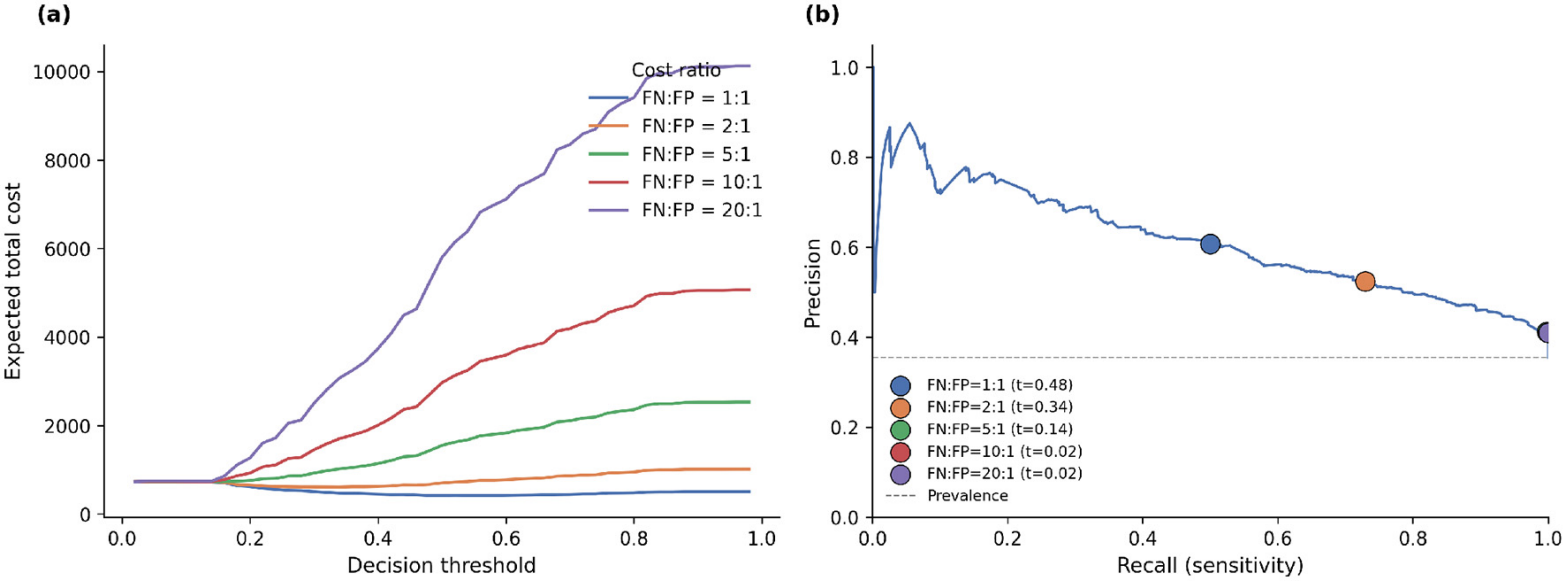
Cost-sensitive threshold sweep for XGBoost on S3.

Because false negatives (missed positive screens, delayed referral) and false positives (unnecessary referral, parental anxiety) carry asymmetric clinical costs, the operating threshold of the *S*_3_ XGBoost model is swept across a range of false-negative-to-false-positive cost ratios. The resulting expected-cost curves and precision–recall trade-offs are shown in Figure 17. Table 8 reports the cost-optimal threshold and the corresponding recall/precision/F1 trade-off. The cost-minimizing threshold drops sharply once the FN:FP ratio exceeds 2:1, prioritizing sensitivity at the expense of precision; thereafter (5:1 and beyond) it stabilizes at a low-threshold, high-recall regime.

**Table 8:** Cost-sensitive operating thresholds for XGBoost on *S*_3_.

FN:FP	Threshold	Recall	Precision	F1
1:1	0.48	0.500	0.607	0.548
2:1	0.34	0.729	0.524	0.610
5:1	0.14	0.998	0.412	0.583
10:1	0.02	1.000	0.410	0.581
20:1	0.02	1.000	0.410	0.581

### 5.5 Sensitivity analysis: disentangling demographic signal from cohort structure

The explainability audit (Section 5.1) shows that the two age features dominate the *S*_3_ model. Because age_group is, by construction, almost perfectly aligned with dataset origin (the value “toddler” occurs only in the Q-CHAT-10 subset, while “child/adolescent/adult” occurs only in the AQ-10 subset) and the two source cohorts carry very different ASD-trait base rates, a model can achieve apparent demographic discrimination simply by inferring *which cohort* a record came from and applying that cohort's prevalence. This is a structural artifact of harmonization rather than a genuine demographic signal, and—although *S*_3_ contains no input to the labeling rule and is therefore leakage-free in the strict sense of Section 3—it can still inflate the reported ceiling. We therefore conduct two sensitivity analyses to quantify how much of the *S*_3_ signal is attributable to cohort structure.

(i) Age-group ablation. We retrain the *S*_3_ models after removing the age_group categorical entirely, retaining only continuous age, sex, jaundice, and family_asd. Because continuous age remains a near-perfect proxy for cohort (toddlers are < 3 years; the AQ-10 cohort is older), this ablation isolates the marginal contribution of the explicit age_group indicator over and above continuous age. (ii) Cohort-stratified audit. We re-run the full *S*_3_ benchmark *separately* within each source cohort—the Q-CHAT-10 toddler subset (*N* = 1054) and the AQ-10 child/adolescent/adult subset (*N* = 6075)—under the same 5-fold stratified protocol. Within a single cohort the cross-cohort prevalence gap is eliminated and the age range is narrow, so the resulting ROC-AUC reflects only within-cohort demographic signal and is the most conservative estimate of genuine demographic discrimination. Results are reported in Table 9.

**Table 9:** Sensitivity analysis on *S*_3_: demographic-feature ablation and cohort stratification.

Configuration	ROC-AUC	Δ vs. unified *S*_3_
Unified *S*_3_ (all demographic features)	0.766 ± 0.005	—
Unified *S*_3_, age_group removed	0.694 ± 0.006	−0.072
Unified *S*_3_, age & age_group removed	0.536 ± 0.013	−0.230
Toddler cohort only (Q-CHAT-10, *S*_3_)	0.615 ± 0.020	−0.151
AQ-10 cohort only (*S*_3_)	0.733 ± 0.012	−0.033

The results in Table 9 confirm the central concern and refine its mechanism. Three findings stand out. First, removing the age_group indicator while retaining continuous age produces a substantial drop (ROC-AUC 0.766 → 0.694, Δ = −0.072), not the negligible change that would be expected if continuous age fully captured cohort membership; the discrete cohort-and-instrument indicator therefore carries strong predictive information in its own right, and is the single most influential demographic feature. Second, removing *both* age features collapses performance to ROC-AUC 0.536, close to the no-skill region, confirming that sex, jaundice, and family_asd carry almost no independent signal: essentially all demographic discriminability on *S*_3_ is age- or cohort-related. Third, and most decisively, the cohort-stratified ceilings lie below the unified-cohort figure—markedly so for the toddler subset (0.615, Δ = −0.151) and modestly for the larger AQ-10 subset (0.733, Δ = −0.033). Because the unified-cohort ROC-AUC (0.766) exceeds *both* within-cohort ceilings, a portion of the apparent demographic discrimination in the merged data must come from the model distinguishing one source cohort from the other and exploiting their differing base rates, rather than from within-cohort demographic risk. Taken together, these results *strengthen* the central conclusion of this audit while correcting its mechanism: demographic metadata alone is a weak predictor of the screening label, the dominant demographic feature is essentially a marker of cohort and instrument provenance, and the unified-cohort ceiling overstates genuine within-cohort discriminability. We therefore treat the unified-cohort *S*_3_ ceiling as an upper bound inflated by cross-cohort structure, and we recommend that audits of merged screening cohorts report cohort-stratified performance as a default rather than relying on a harmonized multi-instrument figure.

## 6 Discussion

Having isolated and quantified leakage (Stages C–L), audited decisions, equity, and calibration (Stages E–A), and measured how much of the leakage-free signal is attributable to cohort structure rather than demographics (Section 5.5), we now interpret what these results imply for the trustworthy use of rule-derived screening labels. The results demonstrate that the near-perfect performance reported across the published Q-CHAT-10 and AQ-10 machine-learning literature is explained, on both datasets, by a single deterministic mechanism: the released label is a downstream function of the questionnaire score, and the score is a deterministic function of the questionnaire items. Cross-tabulation makes the mechanism quantitative: in the toddler subset it is exact (1,054 of 1,054 records), and in the AQ-10 subset it is one-sided and approximate (0 false negatives, 1,076 false positives). Once the questionnaire and score are removed, the predictive signal from demographics alone is modest. Gradient Boosting attains the highest non-leaky ROC-AUC of 0.766 ± 0.005 on *S*_3_, and the three model families converge to within 0.04 ROC-AUC of one another, indicating that the binding constraint is the feature set rather than the model class.

A second, subtler caution applies to the leakage-free figure itself. Section 5.5 shows that the dominant predictor on *S*_3_—continuous age, and the age_group indicator in particular—is partly a marker of *which source cohort* a record belongs to rather than an age-dependent biological signal, because the two instruments carry different base rates (≈69% vs. ≈30% screen-positive) and different positivity thresholds. This is best understood as a form of surveillance/structural bias. The cohort-stratified results therefore provide the more clinically honest estimate of demographic discriminability, and the unified-cohort ceiling of 0.766 should be read as an upper bound inflated by harmonization. We accordingly recommend that audits of merged screening datasets report cohort-stratified performance by default and interpret age effects with explicit reference to instrument provenance.

The findings should not be interpreted as evidence that demographics can diagnose ASD. The released labels are automated screening outcomes derived from parent- or self-report questionnaires and are not linked to DSM-5 diagnosis, ADOS, ADI-R, or physician-confirmed ASD. Therefore, the model predicts a proxy label rather than clinical disease status. External validation against gold-standard clinical assessment is required before any clinical use can be considered.

A natural question is whether the modest ≈0.77 ceiling on *S*_3_ reflects a limitation of the deterministic prompt-structure simulators or a property of the data. We argue it is primarily the latter. On *S*_3_ the model receives only age, sex, jaundice history, and family ASD history; the questionnaire responses that determine the label have been removed by design, so no degree of reasoning sophistication or external knowledge can recover information that is simply absent from the feature vector. Consistent with this, the classical, deep-tabular, and simulator families converge to within 0.04 ROC-AUC (Section 4.3), and within-cohort stratification *lowers* rather than raises the ceiling (Section 5.5). We therefore expect a live-LLM evaluation on identical demographic inputs to perform comparably. A live LLM would, of course, trivially solve *S*_1_ and *S*_2_—but only by re-deriving the labeling rule from the questionnaire items, that is, by reproducing the very leakage this audit is designed to expose.

For researchers who wish to build clinically meaningful models on these populations, the implication is twofold. First, and most importantly, the ceiling reported here is a ceiling on predicting a *proxy* label. The highest-value investment is therefore not a richer feature set paired with the same questionnaire-derived target, but the linkage of new features to gold-standard diagnostic outcomes (DSM-5, ADOS-2, ADI-R); without a validated outcome, even a perfect model would predict only screening status. Recent work that trains on Q-CHAT-derived items yet validates against confirmed clinical diagnoses illustrates this design and reports that compact item subsets retain predictive value against a true diagnostic reference (20). Second, conditional on a valid outcome label, modalities that capture observable behavior—structured developmental history, longitudinal behavioral video, eye-tracking, or vocalization features—are more plausible sources of signal beyond demographics than additional static questionnaire items. We caution, however, that such modalities raise material privacy and consent concerns, especially for young children: behavioral video, voice, and gaze data are biometric and potentially re-identifying, and their responsible collection requires explicit ethics-board oversight, guardian consent, and secure data governance that public questionnaire datasets do not entail. We therefore frame these as directions requiring prospective, ethically governed data collection rather than drop-in fixes, and we deliberately avoid claiming that any single modality will achieve clinically viable performance before it has been validated against a confirmed diagnostic standard.

The subgroup-parity analysis is intentionally descriptive. Several subgroup cells are small (e.g., female toddlers, family-history-positive adolescents), and the observed disparities in predicted positive rates for jaundice and family-history attributes are not unexpected: both attributes raise the prior probability of a positive screening outcome in the underlying instruments. Reporting these disparities as parity statistics rather than hypothesis tests preserves transparency without overclaiming either bias or fairness. The calibration analysis indicates that, on the leakage-free *S*_3_ configuration, the uncalibrated XGBoost model is already well-calibrated (ECE ≈0.02) and that standard interventions such as Platt scaling may worsen rather than improve calibration on this dataset.

Beyond the two-dataset autism-screening case study, Clear-Rd generalizes to other rule-derived screening labels widely used in personalized pediatric and preventive health pipelines, including questionnaire-thresholded depression and anxiety scales (PHQ-9, GAD-7), deterministic risk-stratification scores derived from electronic-health-record variables (CHA_2_DS_2_-VASc, MELD), and wearable-derived activity, sleep, or arrhythmia labels generated by fixed cut-offs. This generalization is supported by prior work in fraud-detection and clinical-sign prediction domains, where imbalanced and rule-adjacent labels have similarly produced inflated performance under conventional evaluation (21, 38). In each setting, the same five stages apply, with only the labeling rule *g* and its input subset *X*_*R*_ instantiated per domain: (C) configure feature sets to expose any deterministic dependence on the labeling rule; (L) quantify performance with stratified cross-validation and non-parametric model comparison; (E) evaluate calibration and decision consequences; (A) audit equity with descriptive parity statistics on disaggregated subgroups; and (R) report a clinical-scope statement aligned with TRIPOD+AI (4). Because each stage produces a named artifact, Clear-Rd outputs can be archived alongside the model card and incorporated directly into clinical-prediction-model documentation.

The demographics-only ceiling of ROC-AUC ≈0.77 should be interpreted in the context of the Q-CHAT-10 instrument's own published screening performance. Allison et al. (13) reported sensitivity and specificity values for the Q-CHAT-10 instrument of approximately 0.91 and 0.89, respectively, in the original validation study, yielding an implied AUC well above 0.90. The demographics-only model therefore falls substantially short of the screening instrument's performance—by more than 0.13 AUC points relative to the instrument's implied discriminative ability. This gap reinforces the central clinical-scope conclusion of this paper: demographic metadata alone is a weak predictor of the questionnaire-derived label, and no demographics-only model trained on these datasets can substitute for, or claim equivalence with, the screening instruments themselves.

## 7 Limitations

This study has six limitations. First, the outcome is a screening-rule label rather than a clinical ASD diagnosis. Second, the evaluation is within-cohort and lacks external validation against an independent population or a gold-standard clinical reference standard. Third, the schema-harmonization step required dropping the ethnicity and test-administrator variables, which are present only in the toddler subset; as a consequence the audit cannot evaluate ethnic bias or report ethnicity-stratified performance—an important fairness dimension for any deployable screening model—and the subgroup-parity analysis is restricted to the attributes (sex, jaundice, family ASD history, age group) common to both subsets. Fourth, the four prompt-structure simulators preserve prompting *structure* but not the external knowledge or learned parameters of a live large language model; their convergence to within 0.04 ROC-AUC of the classical and deep-tabular families is informative but does not by itself prove that a live large-language-model evaluation could recover no additional signal, although the demographics-only information-bottleneck argument in Section 6 makes such a gain unlikely on *S*_3_ inputs. Fifth, the unified cohort is the union of two convenience samples rather than a population-representative cohort, and the absolute calibration and subgroup-parity findings should not be extrapolated to broader pediatric or adult populations without an independent validation study. Sixth, because the cohort merges a toddler-specific instrument with a lifespan instrument, continuous age and the age_group indicator are confounded with source-cohort membership and its associated base-rate and threshold differences; the sensitivity analysis in Section 5.5 quantifies this structural confound but cannot fully remove it, and we recommend that future audits evaluate age-stratified or single-instrument cohorts separately rather than relying on a harmonized multi-instrument cohort.

## 8 Conclusion

This paper introduces Clear-Rd (*Clinical Leakage Evaluation and Audit Routine for Rule-Derived labels*), a five-stage trustworthy-AI evaluation framework—Configure, Lift quantify, Evaluate decisions, Audit equity, Report scope—and instantiates it on a unified cohort built from two widely-used public autism-screening datasets, Q-CHAT-10 and AQ-10. The instantiation shows that the near-perfect performance reported in prior machine-learning studies on these datasets is largely attributable to deterministic label leakage from the questionnaire score and items; cross-tabulation makes the leakage quantitative on both datasets. In the leakage-free demographics-only configuration, Gradient Boosting attains ROC-AUC = 0.766 ± 0.005 under 5-fold stratified cross-validation, and classical machine-learning, deep tabular, and prompt-structure simulator families converge to within 0.04 ROC-AUC, indicating that the binding constraint is the feature set rather than the model class. The framework's audit stages further show that uncalibrated probabilities are already well-calibrated on this configuration, that subgroup disparities track attributes that are correlated with the rule-derived label by construction, and that cost-sensitive operating thresholds change sharply once the false-negative-to-false-positive ratio exceeds 2:1. As stage R deliverable: no evaluated model is deployable without external validation against gold-standard ASD diagnoses (DSM-5, ADOS, ADI-R, or physician confirmation). Trustworthy personalized screening AI requires externally validated labels, prospective evaluation, and subgroup-aware reporting; Clear-Rd provides a readily adaptable template for any rule-derived medical label and a foundation for delivering these assurances within personalized pediatric and preventive health pipelines. This work aligns with the TRIPOD+AI reporting framework (4); a completed TRIPOD+AI checklist is provided as Supplementary material 1.

## 9 Reproducibility details

All experiments use a fixed random seed of 42 throughout: for stratified fold assignment in 5-fold cross-validation, for the validation split inside the deep-tabular trainers, and for any internal model-side randomness. The classical-ML benchmark uses scikit-learn estimators with library defaults and the standard XGBoost and LightGBM Python interfaces. The two deep-tabular networks (MLP-Emb and FT-Transformer) are trained with early stopping. The four prompt-structure simulators (P1–P4) are deterministic NumPy procedures that take the training fold as input and return per-record positive-class probabilities for the test fold. The Friedman omnibus test is computed on per-fold ROC-AUC ranks; the Nemenyi *post-hoc* critical-difference diagram uses the scikit-*post hocs* implementation. End-to-end runtime on a single CPU is approximately 20 min.

## Data Availability

Publicly available datasets were analyzed in this study. This data can be found here: https://www.kaggle.com/datasets/fabdelja/autism-screening-for-toddlers ; https://www.kaggle.com/datasets/fabdelja/asd-screening-data-toddler-child-adoles-adult.

## References

[1] Rajpurkar P Chen E Banerjee O Topol EJ. AI in health and medicine. Nat Med. (2022) 28:31–8. doi: 10.1038/s41591-021-01614-0 35058619

[2] Ben Ammar B Salem A Ben Said M Ben Aouicha M. Machine learning models for early prediction of COVID-19 infections based on clinical signs. SN Comput Sci. (2024) 5:158. doi: 10.1007/s42979-023-02489-3

[3] Chen RJ Wang JJ Williamson DFK Chen TY Lipkova J Lu MY . Algorithmic fairness in artificial intelligence for medicine and healthcare. Nat Biomed Eng. (2023) 7:719–42. doi: 10.1038/s41551-023-01056-8 37380750 PMC10632090

[4] Collins GS Moons KGM Dhiman P Riley RD Beam AL Van Calster B . TRIPOD+AI statement: updated guidance for reporting clinical prediction models. BMJ. (2024.385:e078378. doi: 10.1136/bmj-2023-078378 38626948 PMC11019967

[5] Ricci Lara MA Echeveste R Ferrante E. Addressing fairness in artificial intelligence for medical imaging. Nat Commun. (2022) 13:4581. doi: 10.1038/s41467-022-32186-3 35933408 PMC9357063

[6] Kaufman S Rosset S Perlich C Stitelman O. Leakage in data mining: formulation, detection, and avoidance. ACM Trans Knowl Discov Data. (2012) 6:1–21. doi: 10.1145/2382577.2382579

[7] Kapoor S Narayanan A. Leakage and the reproducibility crisis in machine-learning-based science. Patterns. (2023) 4:100804. doi: 10.1016/j.patter.2023.100804 37720327 PMC10499856

[8] Vandewiele G Dehaene I Kovács G Sterckx L Janssens O Ongenae F . Overly optimistic prediction results on imbalanced data. Artif Intell Med. (2021) 111:101987. doi: 10.1016/j.artmed.2020.101987 33461687

[9] Saeb S Lonini L Jayaraman A Mohr DC Kording KP. The need to approximate the use-case in clinical machine learning. GigaScience. (2017) 6:gix019. doi: 10.1093/gigascience/gix019 28327985 PMC5441397

[10] Maenner MJ. Prevalence and characteristics of autism spectrum disorder among children aged 8 years. MMWR Surveill Summ. (2023) 72:1–14. doi: 10.15585/mmwr.ss7202a1 36952288 PMC10042614

[11] Zwaigenbaum L Bauman ML Choueiri R Fein D Kasari C Pierce K . Early identification and interventions for autism spectrum disorder. Pediatrics. (2015) 136(Suppl. 1):S1–S9. doi: 10.1542/peds.2014-3667B 26430167 PMC9923899

[12] Pierce K Carter C Weinfeld M Desmond J Hazin R Bjork R . Detecting, studying, and treating autism early: the one-year well-baby check-up approach. J Pediatr. (2011) 159:458–65. doi: 10.1016/j.jpeds.2011.02.036 21524759 PMC3157595

[13] Allison C Baron-Cohen S Wheelwright S Charman T Richler J . The Q-CHAT (Quantitative Checklist for Autism in Toddlers). J Autism Dev Disord. (2008) 38:1414–25. doi: 10.1007/s10803-007-0509-7 18240013

[14] Thabtah F. An accessible and efficient autism screening method for behavioural data and predictive analyses. Health Informat J. (2019) 25:1739–55. doi: 10.1177/1460458218796636 30230414

[15] Akter T Khan MI Ali MH Satu MS Uddin MJ Moni MA. Improved machine learning based classification model for early autism detection. In: Proc. 2nd Int. Conf. Robotics, Electrical and Signal Processing Techniques (ICREST) (2021). Dhaka: IEEE. p. 742–7. doi: 10.1109/ICREST51555.2021.9331013

[16] Vakadkar K Purkayastha D Krishnan D. Detection of autism spectrum disorder in children using machine learning techniques. SN Comput Sci. (2021) 2:386. doi: 10.1007/s42979-021-00776-5 34316724 PMC8296830

[17] Hyde KK Novack MN LaHaye N Parlett-Pelleriti C Anden R Dixon DR . Applications of supervised machine learning in autism spectrum disorder research: a review. Rev J Autism Dev Disord. (2019) 6:128–46. doi: 10.1007/s40489-019-00158-x

[18] Song D. Y., Kim, S. Y., Bong, G., Kim, J. M., and Yoo, H. J. (2019). The use of artificial intelligence in screening and diagnosis of autism spectrum disorder: a literature review. J. Korean Acad. Child Adolesc. Psychiatry. 30, 145. doi: 10.5765/jkacap.190027 32595335 PMC7298904

[19] Tartarisco G Cicceri G Di Pietro D Leonardi E Aiello S Marino F . Use of machine learning to investigate the quantitative checklist for autism in toddlers (Q-CHAT) towards early autism screening. Diagnostics. (2021) 11:574. doi: 10.3390/diagnostics11030574 33810146 PMC8004748

[20] Sollis LJ Wall DP Washington PY. Compact subsets of autism screening items predict clinical diagnoses with a machine learning analysis of the QCHAT-10. Sci Rep. (2025) 15:39091. doi: 10.1038/s41598-025-26131-9 41203747 PMC12594979

[21] Rubaidi ZS Ben Ammar B Ben Aouicha M. Fraud detection using large-scale imbalance dataset. Int J Artif Intell Tools. (2022) 31:2250037. doi: 10.1142/S0218213022500373

[22] Lundberg SM Lee S-I. A unified approach to interpreting model predictions. In: Proc. NeurIPS. Red Hook, NY: Curran Associates, Inc. (2017).

[23] Ribeiro MT Singh S Guestrin C. Why should I trust you? Explaining the predictions of any classifier. In: *Proc. ACM SIGKDD*. New York, NY: Association for Computing Machinery (ACM) (2016). p. 1135–44. doi: 10.1145/2939672.2939778

[24] Niculescu-Mizil A Caruana R. Predicting good probabilities with supervised learning. In: Proc. ICML (2005). New York, NY: Association for Computing Machinery (ACM), p. 625–32. doi: 10.1145/1102351.1102430

[25] Guo C Pleiss G Sun Y Weinberger KQ. On calibration of modern neural networks. In: Proc. ICML. Sydney, NSW: PMLR. (2017). p. 1321–30.

[26] Hardt M Price E Srebro N. Equality of opportunity in supervised learning. In: Proc. NeurIPS. Red Hook, NY: Curran Associates, Inc. (2016).

[27] Agarwal A Beygelzimer A Dudik M Langford J Wallach H. A reductions approach to fair classification. In: Proceedings of the 35th international conference on machine learning. Stockholm: Proceedings of Machine Learning Research (PMLR) (2018). p. 60–9.

[28] Saleiro P Kuester B Stevens A Anisfeld A Hinkson L London J . Aequitas: a bias and fairness audit toolkit. arXiv [Preprint] *arXiv:1811.05577*. (2018).

[29] Bellamy RKE Dey K Hind M Hoffman SC Houde S Kannan K . AI Fairness 360: an extensible toolkit for detecting and mitigating algorithmic bias. IBM J Res Dev. (2019) 63:1–4. doi: 10.1147/JRD.2019.2942287

[30] Wexler J., Pushkarna, M., Bolukbasi, T., Wattenberg, M., Viégas, F., and Wilson, J. (2019). The what-if tool: Interactive probing of machine learning models. IEEE Trans. Vis. Comput. Graph. 26, 56–65. doi: 10.1109/TVCG.2019.2934619 31442996

[31] Demšar J. (2006). Statistical comparisons of classifiers over multiple data sets. J. Mach. Learn. Res. 7, 1–30. doi: 10.5555/1248547.1248548

[32] Grinsztajn L Oyallon E Varoquaux G. Why do tree-based models still outperform deep learning on tabular data? In: Proceedings of the NeurIPS datasets benchmarks track. Red Hook, NY: Curran Associates, Inc. (2022). doi: 10.52202/068431-0037

[33] Shwartz-Ziv R Armon A. Tabular data: deep learning is not all you need. Inf Fusion. (2022) 81:84–90. doi: 10.1016/j.inffus.2021.11.011

[34] Rubaidi ZS Ben Ammar B Ben Aouicha M. Comparative data oversampling techniques with deep learning algorithms for credit card fraud detection. In: Intelligent Systems Design and Applications (ISDA 2022), Lecture Notes in Networks and Systems. Cham: Springer (2023), 646.

[35] Hanley JA McNeil BJ. The meaning and use of the area under a receiver operating characteristic curve. Radiology. (1982) 143:29–36. doi: 10.1148/radiology.143.1.7063747 7063747

[36] Saito T Rehmsmeier M. The precision-recall plot is more informative than the ROC plot when evaluating binary classifiers on imbalanced datasets. PLoS ONE. (2015) 10:e0118432. doi: 10.1371/journal.pone.0118432 25738806 PMC4349800

[37] DeLong ER DeLong DM Clarke-Pearson DL. Comparing the areas under two or more correlated receiver operating characteristic curves: a nonparametric approach. Biometrics. (1988) 44:837–45. doi: 10.2307/2531595 3203132

[38] Ben Ammar B., and Rubaidi, Z. S. (2026). Integrating sequential hybrid oversampling with decision-theoretic threshold design for credit risk assessment. Mathematics 14:1467. doi: 10.3390/math14091467

