# Supplementary material for: Label leakage unmasked: a trustworthy-AI audit of autism screening models using the CLEAR-RD framework
Source: Front Public Health. 2026 Jun 30;14:1881829. doi: 10.3389/fpubh.2026.1881829 (PMC13365301; doi:10.3389/fpubh.2026.1881829)
Supplement: Supplementary file 1 [file Supplementary_File_1.docx]

**Supplementary Material 1 — TRIPOD+AI Reporting Checklist**

|  | **Item** | **D/E** | **TRIPOD+AI checklist item** | **Where reported in CLEAR-RD** |
| --- | --- | --- | --- | --- |
| **Title** | 1 | D;E | Identify the study as developing or evaluating a multivariable prediction model, the target population, and the outcome to be predicted. | Title identifies the work as an evaluation and audit of machine-learning autism-screening prediction models (Q-CHAT-10 toddler and AQ-10 child/adolescent/adult), predicting the screening-positive class. |
| **Abstract** | 2 | D;E | See TRIPOD+AI for Abstracts checklist (completed below). | Abstract — the structured abstract reports the objective, the datasets, the leakage finding, the leakage-free performance, the audit components (calibration, subgroup parity, cost-sensitivity), and the clinical-scope limitation. |
| **Background** | 3a | D;E | Explain the healthcare context (diagnostic/prognostic) and rationale for developing or evaluating the model, including references to existing models. | Introduction — diagnostic screening context and the rationale for auditing prior published machine-learning autism-screening models for label leakage and methodological integrity; existing models referenced. |
| **Background** | 3b | D;E | Describe the target population, the intended purpose of the model in the care pathway, and its intended users. | Introduction — target population (individuals undergoing autism screening across the toddler, child, adolescent, and adult instruments); intended purpose as screening triage; intended users (clinicians and screening practitioners). |
| **Background** | 3c | D;E | Describe any known health inequalities between sociodemographic groups. | Introduction / Discussion — known demographic disparities in autism screening and access are discussed. |
| **Objectives** | 4 | D;E | Specify the study objectives, including whether the study describes development or validation of a model (or both). | Introduction — study objectives stated: an evaluation and audit of previously reported models using the CLEAR-RD five-stage framework, benchmarking sixteen models across three families. |
| **Data** | 5a | D;E | Describe the sources of data separately for development and evaluation datasets, the rationale, and representativeness. | Methods (Datasets) — two publicly available autism-screening datasets, Q-CHAT-10 (toddler) and AQ-10 (child/adolescent/adult); rationale and representativeness limitations described. |
| **Participants** | 6a | D;E | Specify key elements of the study setting and the number and location of centres. | Methods (Datasets) — setting described as publicly available, de-identified screening data; a single data source per dataset; no multi-centre design. |
| **Participants** | 6b | D;E | Describe the eligibility criteria for study participants. | Methods (Datasets) — eligibility comprised records with complete screening responses in the public datasets. |
| **Participants** | 6c | D;E | Give details of any treatments received and how handled, if relevant. | Not applicable — the screening datasets contain no treatment information. |
| **Data preparation** | 7 | D;E | Describe any data pre-processing and quality checking, including whether similar across sociodemographic groups. | Methods — data pre-processing and quality checking are described, including the label-leakage audit central to the CLEAR-RD framework. |
| **Outcome** | 8a | D;E | Clearly define the predicted outcome and time horizon, how/when assessed, rationale, and whether assessment is consistent across groups. | Methods — the predicted outcome is the screening-positive label; the label is derived from the screening-instrument scoring rule, which is the central leakage concern examined. |
| **Outcome** | 8b | D;E | If outcome assessment requires subjective interpretation, describe assessors' qualifications and demographics. | Not applicable — outcome labels are derived deterministically from the questionnaire scoring rule and do not involve subjective assessment. |
| **Outcome** | 8c | D;E | Report any actions to blind assessment of the outcome. | Not applicable — secondary analysis of pre-labelled public data; no prospective outcome assessment was conducted. |
| **Predictors** | 9a | D | Describe the choice of initial predictors and any pre-selection before model building. | Methods — predictors comprise the screening questionnaire items and demographic fields, consistent with the replicated studies. |
| **Predictors** | 9b | D;E | Clearly define all predictors, including how and when measured (and any blinding). | Methods — all predictors are defined, with measurement at the time of screening. |
| **Predictors** | 9c | D;E | If predictor measurement requires subjective interpretation, describe assessors' qualifications and demographics. | Not applicable — predictors are fixed self- or caregiver-reported questionnaire responses requiring no subjective assessor interpretation. |
| **Sample size** | 10 | D;E | Explain how the study size was arrived at and justify sufficiency; include any sample size calculation. | Methods — the full available public cohort was used; no formal a priori sample-size calculation was performed, which is noted as a limitation. |
| **Missing data** | 11 | D;E | Describe how missing data were handled; give reasons for omitting any data. | Methods — the handling of missing or incomplete records is described. |
| **Analytical methods** | 12a | D | Describe how the data were used (development/evaluation), including any partitioning, considering sample size requirements. | Methods — model performance was estimated using 5-fold cross-validation. |
| **Analytical methods** | 12b | D | Describe how predictors were handled in the analyses (functional form, rescaling, transformation, standardisation). | Methods — predictor encoding and handling are described as implemented in the modelling pipeline. |
| **Analytical methods** | 12c | D | Specify the model type, rationale, all model-building steps including hyperparameter tuning, and internal validation. | Methods — three model families were evaluated: ten classical machine-learning estimators (including Gradient Boosting, XGBoost, LightGBM, Random Forest, Decision Tree, SVM-RBF, k-NN), two deep-tabular networks (MLP-Emb, FT-Transformer), and four deterministic prompt-structure simulators (P1–P4); classical models used default hyperparameters, and internal validation was by 5-fold cross-validation. |
| **Analytical methods** | 12d | D;E | Describe if/how heterogeneity in parameter values and performance was handled/quantified across clusters. | Not applicable — the data have no clustered or multi-centre structure. |
| **Analytical methods** | 12e | D;E | Specify all measures and plots used (and rationale) to evaluate performance and, if relevant, compare models. | Methods — performance was evaluated using ROC-AUC (primary), with PR-AUC, F1, and balanced accuracy; models were compared with the Friedman test and a Nemenyi critical-difference diagram, and BCa bootstrap 95% confidence intervals and DeLong tests were reported on the held-out set. |
| **Analytical methods** | 12f | E | Describe any model updating (e.g., recalibration) arising from evaluation. | Not applicable — no model updating or recalibration was performed. |
| **Analytical methods** | 12g | E | For model evaluation, describe how predictions were calculated (formula, code, object, API). | Methods / replication package — model predictions are reproduced from the released code. |
| **Class imbalance** | 13 | D;E | If class imbalance methods were used, state why and how, and any recalibration. | No class-imbalance correction or resampling was applied. |
| **Fairness** | 14 | D;E | Describe any approaches used to address model fairness and their rationale. | Methods (Fairness) — the fairness-audit approach is described and differentiated from general-purpose toolkits (Aequitas, IBM AI Fairness 360, and Google PAIR). |
| **Model output** | 15 | D | Specify the model output (e.g., probabilities, classification) and rationale for any classification thresholds. | Methods — the model output is a predicted probability with threshold-based classification, consistent with the replicated studies. |
| **Training vs evaluation** | 16 | D;E | Identify any differences between development and evaluation data in setting, eligibility, outcome, and predictors. | Methods — development and evaluation are performed on the same datasets under cross-validation; there is no separate external evaluation cohort, which is noted as a limitation. |
| **Ethical approval** | 17 | D;E | Name the IRB/ethics committee and describe consent or waiver. | Ethics — secondary analysis of publicly available, de-identified data; institutional ethics approval was not required. |
| **Funding** | 18a | D;E | Give the source of funding and the role of the funders. | Declarations — No specific funding was received for this work. |
| **Conflicts of interest** | 18b | D;E | Declare any conflicts of interest and financial disclosures for all authors. | Declarations — The authors declare no competing interests. |
| **Protocol** | 18c | D;E | Indicate where the protocol can be accessed or state none was prepared. | Declarations — A study protocol was not prepared. |
| **Registration** | 18d | D;E | Provide study registration information or state not registered. | Declarations — The study was not registered. |
| **Data sharing** | 18e | D;E | Provide details of the availability of the study data. | Open science — both datasets are publicly available; their sources are cited. |
| **Patient & public involvement** | 19 | D;E | Provide details of any patient and public involvement, or state none. | Declarations — There was no patient or public involvement in this study. |
| **Participants (results)** | 20a | D;E | Describe the flow of participants, including numbers with/without outcome; a diagram may help. | Results — participant counts per dataset and class are reported; a workflow diagram is provided. |
| **Participants (results)** | 20b | D;E | Report characteristics overall and by source/setting (key dates, predictors, demographics, sample size, events, missing data). | Results (Table) — participant and predictor characteristics are reported per dataset. |
| **Participants (results)** | 20c | E | For evaluation, compare distribution of important predictors with the development data. | Not applicable — no separate external evaluation cohort; the same data are used under cross-validation. |
| **Model development** | 21 | D;E | Specify the number of participants and outcome events in each analysis. | Results — the number of participants and outcome events in each analysis is reported. |
| **Model specification** | 22 | D | Provide details of the full prediction model to allow predictions in new individuals and third-party evaluation, including access restrictions. | Replication package — the released model and code enable third-party evaluation. |
| **Model performance** | 23a | D;E | Report performance estimates with confidence intervals, including key subgroups; consider plots. | Results — a ROC-AUC of 0.766 ± 0.005 from 5-fold cross-validation is reported on the leakage-free configuration, with BCa bootstrap 95% confidence intervals for the best model per family (e.g., 0.759, 95% CI 0.734–0.783). |
| **Model performance** | 23b | D;E | If examined, report heterogeneity in performance across clusters. | Not applicable — no clustered structure was examined. |
| **Model updating** | 24 | E | Report results from any model updating. | Not applicable — no model updating was performed. |
| **Interpretation** | 25 | D;E | Give an overall interpretation of the main results, including fairness, in context of objectives and prior studies. | Discussion — an overall interpretation of the discrimination signal and leakage findings relative to prior studies is given, including fairness considerations. |
| **Limitations** | 26 | D;E | Discuss limitations (non-representative sample, sample size, overfitting, missing data) and their effects. | Discussion — limitations include dataset representativeness, the modest discrimination signal, single-source data, and the absence of external validation. |
| **Usability** | 27a | D | Describe how poor-quality or unavailable input data should be assessed/handled at implementation. | Discussion — the implications of poor-quality or unavailable input data for implementation are discussed. |
| **Usability** | 27b | D | Specify whether users must interact in handling input/use of the model and the expertise required. | Discussion — the user interaction and expertise required to use the model are discussed. |
| **Usability** | 27c | D;E | Discuss next steps for future research, with a view to applicability and generalisability. | Conclusion — next steps for leakage-aware auditing and external validation are outlined. |

D = development items; E = evaluation items; D;E = both.
